# Sulfation and amidinohydrolysis in the biosynthesis of giant linear polyenes

**DOI:** 10.3762/bjoc.13.238

**Published:** 2017-11-13

**Authors:** Hui Hong, Markiyan Samborskyy, Katsiaryna Usachova, Katharina Schnatz, Peter F Leadlay

**Affiliations:** 1 Department of Biochemistry, University of Cambridge, Cambridge CB2 1GA, UK

**Keywords:** amidinohydrolase, clethramycin, mediomycin, polyketide synthase, sulfotransferase

## Abstract

Clethramycin from *Streptomyces malaysiensis* DSM4137, and mediomycins (produced together with clethramycin from *Streptomyces mediocidicus*), are near-identical giant linear polyenes apparently constructed from, respectively, a 4-guanidinobutanoate or 4-aminobutanoate starter unit and 27 polyketide extender units, and bearing a specific *O*-sulfonate modification at the C-29 hydroxy group. We show here that mediomycins are actually biosynthesised not by use of a different starter unit but by direct late-stage deamidination of (desulfo)clethramycin. A gene (*slf*) encoding a candidate sulfotransferase has been located in both gene clusters. Deletion of this gene in DSM4137 led to accumulation of desulfoclethramycin only, instead of a mixture of desulfoclethramycin and clethramycin. The mediomycin gene cluster does not encode an amidinohydrolase, but when three candidate amidinohydrolase genes from elsewhere in the *S. mediocidicus* genome were individually expressed in *Escherichia coli* and assayed, only one of them (*medi4948*), located 670 kbp away from the mediomycin gene cluster on the chromosome, catalysed the removal of the amidino group from desulfoclethramycin. Subsequent cloning of *medi4948* into DSM4137 caused mediomycins A and B to accumulate at the expense of clethramycin and desulfoclethramycin, respectively, a rare case where an essential biosynthetic gene is not co-located with other pathway genes. Clearly, both desulfoclethramycin and clethramycin are substrates for this amidinohydrolase. Also, purified recombinant sulfotransferase from DSM4137, in the presence of 3'-phosphoadenosine-5'-phosphosulfate as donor, efficiently converted mediomycin B to mediomycin A in vitro. Thus, in the final steps of mediomycin A biosynthesis deamidination and sulfotransfer can take place in either order.

## Introduction

**Scheme 1:**
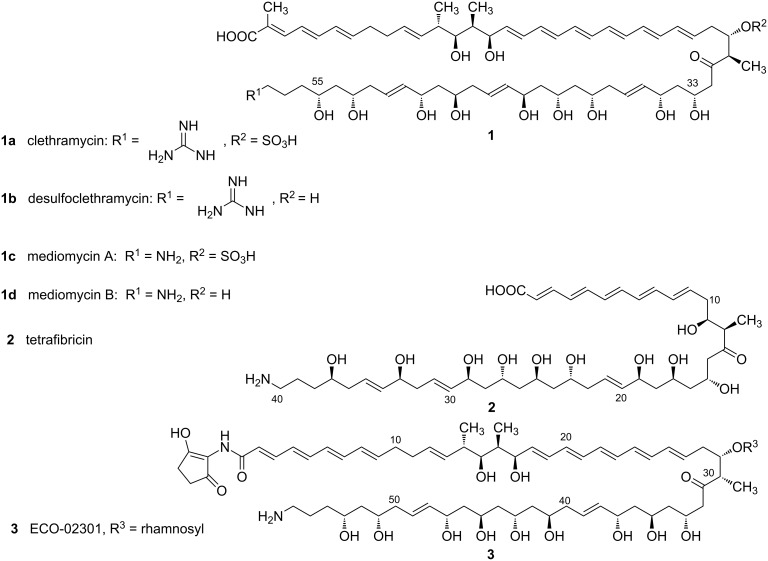
The structures of clethramycin, mediomycins and related linear polyenes.

Bacterial modular polyketide synthases (PKSs) follow an assembly-line paradigm for enzyme catalysis, in which each round of chain extension requires a different set, or module, of enzymatic activities [1–4]. Among the more remarkable natural products derived by this pathway is the giant linear polyene clethramycin (**1a**, Scheme 1), originally isolated as an antifungal and as an inhibitor of pollen tube outgrowth from a plant-associated *Streptomyces hygroscopicus* strain [5]. Clethramycin (together with desulfoclethramycin (**1b**)) is also a product of the prolific strain *Streptomyces malaysiensis* DSM4137 (formerly *Streptomyces violaceusniger* DSM4137) [6] and it co-occurs with the closely-related antifungal mediomycins A (**1c**) and B (**1d**, Scheme 1) in *Streptomyces mediocidicus* ATCC 23936 [7]. Mediomycins are also produced by *Streptomyces blastmyceticus* [8]. The genes for the assembly-line PKS for mediomycin from *S. blastmyceticus* have been recently reported, encoding a separate extension module for all 27 cycles of chain extension [8]. These systems are attractive targets for knowledge-based engineering to produce novel antifungal compounds. A member of this class of polyketides, tetrafibricin from *Streptomyces neyagawaensis* (Scheme 1), is a potent inhibitor of the fibrinogen receptor, which suggests an even wider potential utility for such compounds [8–9].

We have previously shown that in the biosynthesis of giant macrocyclic antifungal polyketides (so-called marginolactones) compounds bearing a terminal amino moiety are formed by specific final-stage deamidination of a precursor bearing a guanidino substituent at this position [10]. The aim of the present study was to test whether the same "protective group" strategy is operating in the biosynthesis of the giant linear mediomycins. If true, then mediomycin A formation involves quite distinctive late-stage processing: both an *O*-sulfonation step and a deamidination step. *O*-Sulfonation in particular is a rare and interesting modification seen in diverse microbial natural products (Scheme S1, Supporting Information File 1) including the non-glycosylated teicoplanin-related antibacterial A47934 [11], the engineered trisubstituted sulfoteicoplanin aglycone G [12], the echinocandin-like FR901379 [13]; and the sulfated carbapenem MM4550 [14]. A specific amidinohydrolase has been found encoded in the respective biosynthetic gene clusters for the marginolactone antibiotics primycin and desertomycin [10] but not in the reported cluster for mediomycin in *S. blastmyceticus* [8], so the enzyme hypothesised to be responsible for this step in mediomycin biosynthesis has not been identified until now. We present here the characterisation of the *med* biosynthetic gene cluster, the successful identification of a specific mediomycin amidinohydrolase encoded at a location on the genome remote from the *med* cluster, and genetic and biochemical evidence for the respective roles played by these sulfotransferase and amidinohydrolase enzymes in the production of mediomycins.

## Results and Discussion

**Scheme 2:**
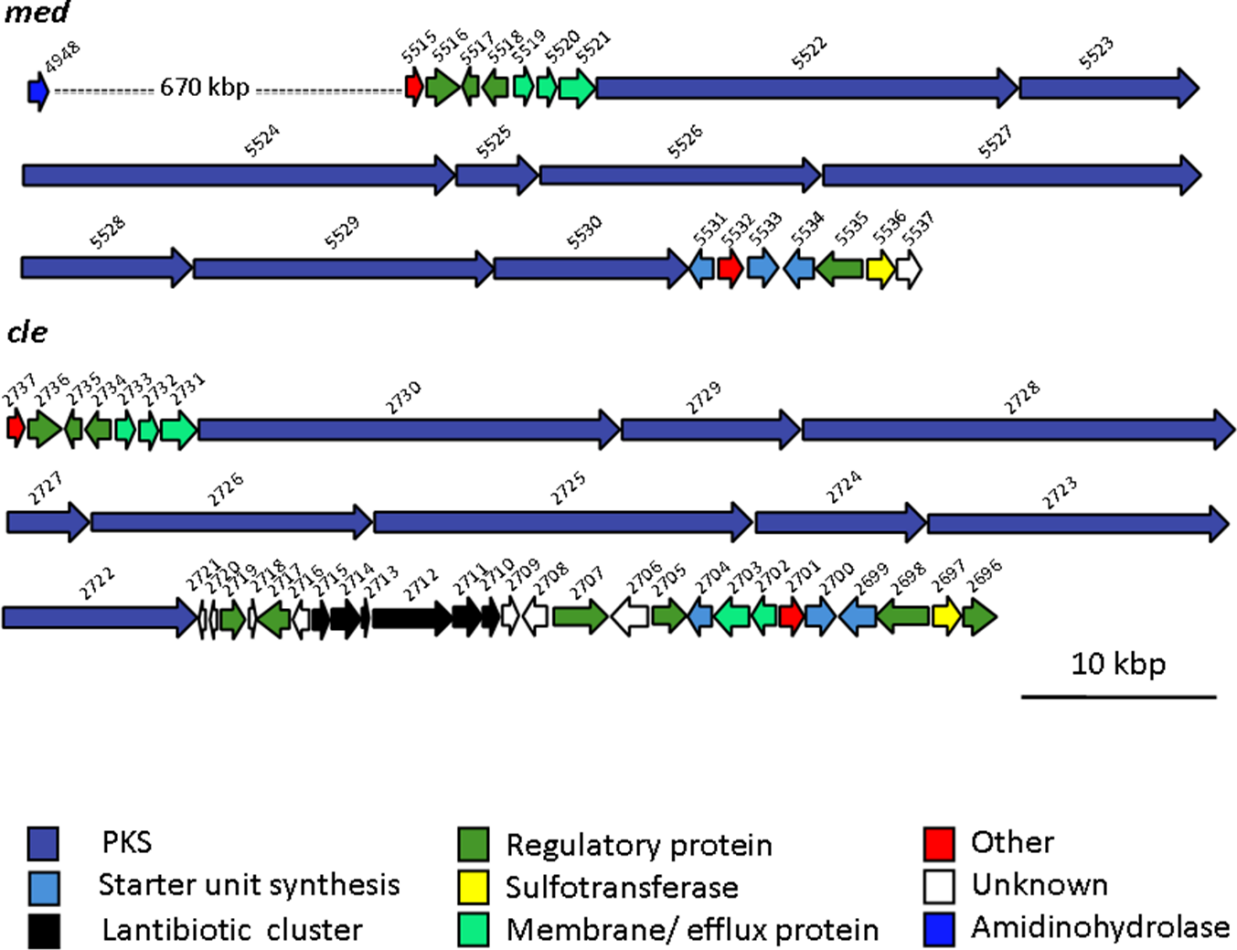
The biosynthetic gene clusters for mediomycin (*med*) from *Streptomyces mediocidicus* and clethramycin (*cle*) from *S. malaysiensis* DSM4137. The numbers refer to the position of each orf in the genome sequence.

We have previously [10] sequenced the biosynthetic gene cluster for clethramycin from *S. malaysiensis* DSM4137 (*cle*); and we have now also sequenced the cluster for mediomycins A and B from *S. mediocidicus* (*med*), by whole-genome sequencing of the strain. Clethramycin and desulfoclethramycin were not previously known to be produced by *S. malaysiensis* DSM4137, but the identity of these metabolites in cell extracts was readily confirmed by high-resolution mass analysis (Figure S1, Supporting Information File 1). The properties of the genes and proteins in the *med* cluster and *cle* cluster are summarised in Table S4 (Supporting Information File 1). Both gene clusters encode a giant modular PKS housing 27 extension modules, distributed across nine PKS subunits (Scheme 2). This is exactly the number of extension modules predicted on the basis of the known structures of **1a**–**d**. Detailed comparison of our amino acid sequences for the Cle PKS [10] and the Med PKS with the sequence recently reported for the Med PKS of *S. blastmyceticus* [8] showed in each case a high degree of amino acid sequence identity across the entire PKS (≈96% identity) and essentially perfect conservation of sequence in each of the characteristic active site sequence motifs for the ketosynthase (KS), acyltransferase (AT), ketoreductase (KR), dehydratase (DH), and enoylreductase (ER) domains of each of the 27 extension modules, including the newly-recognised YGP motif of active DH domains [8] between the three PKS multienzymes. The stereochemistry of **1a**–**d** shown in Scheme 1 is that predicted from the biosynthetic analysis; and is in full agreement with the experimentally-determined relative configuration of stereocentres in mediomycin [7–8]. The flanking genes are also for the most part near-identical, including a sulfotransferase (*slf*) gene that appears to mark one boundary of the cluster. However, in the *cle* cluster there are additional putative regulatory and export genes, as well as, remarkably, a set of six genes interpolated into the cluster that are predicted to govern lantibiotic biosynthesis (Scheme 2).

**Figure 1:**
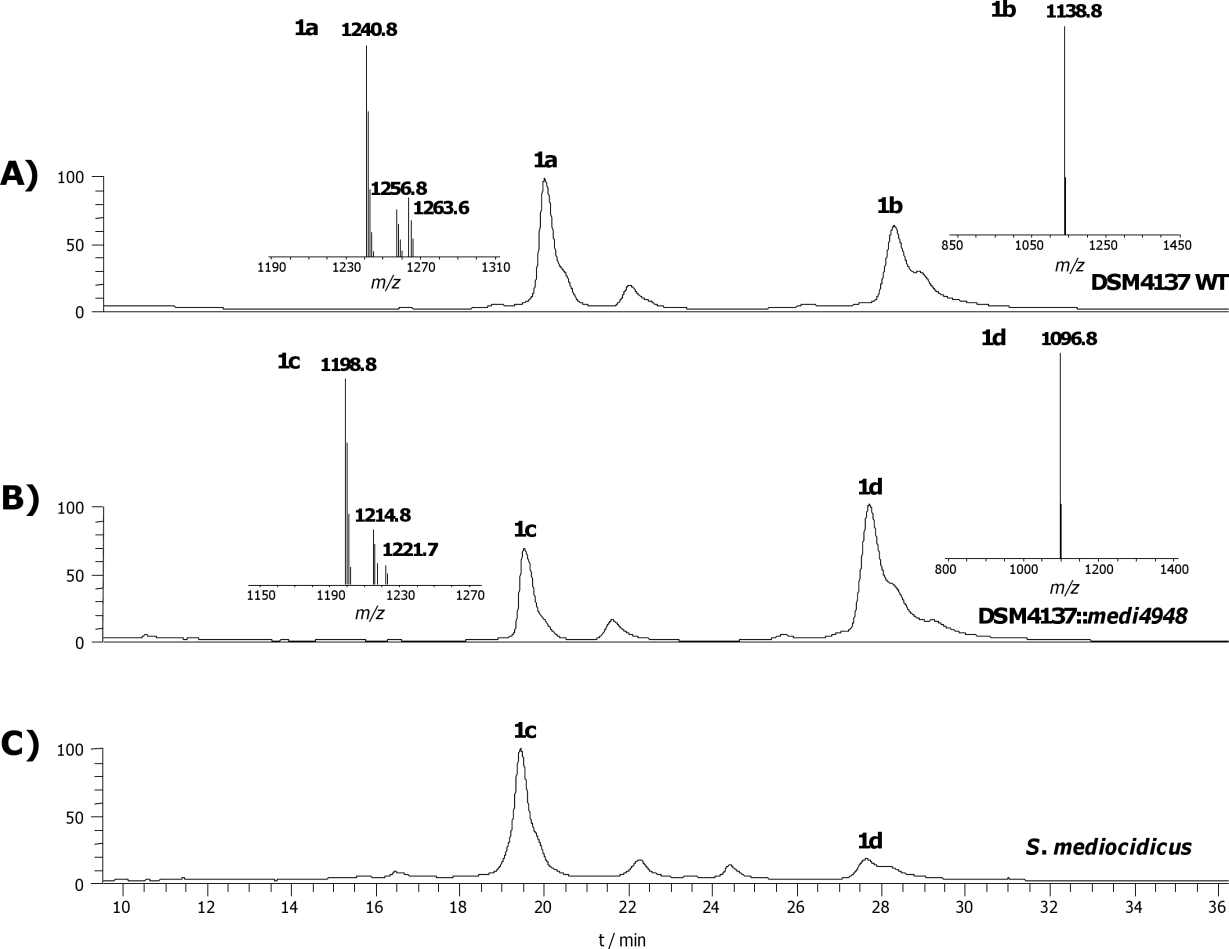
HPLC–UV–MS analysis of polyenes. A) LC–UV (360 nm) trace of mycelium methanol extract from DSM4137 wild type, showing the production of clethramycin (**1a**) and desulfoclethramycin (**1b**) at *m/z* 1240.8 ([M + Na]^+^) and 1138.8 ([M + H]^+^) , respectively. B) LC–UV (360 nm) trace of mycelium methanol extract from DSM4137 complemented with amidinohydrolase gene *medi4948* from *S. mediocidicus*, showing clethramycin (**1a**) and desulfoclethramycin (**1b**) are fully converted to the corresponding amino forms, mediomycin A (**1c**) and mediomycin B (**1d**) at *m/z* 1198.8 ([M + Na]^+^) and 1096.8 ([M + H]^+^), respectively. C) LC–UV (360 nm) trace of mycelium methanol extract from *S. mediocidicus*, showing the production of mediomycin A (**1c**) and mediomycin B (**1d**).

The key difference expected between the *cle* and the *med* clusters was the presence of an essential amidinohydrolase gene, encoding an enzyme that would act at a late stage in the pathway to unmask the primary amino group of the mediomycins, as we have previously described for the biosynthesis of aminomarginolactone antibiotics [10]. However, careful scrutiny of the open reading frames flanking the PKS in the *med* biosynthetic gene cluster failed to reveal any whose product could be plausibly construed to be an amidinohydrolase. The reported *S. blastmyceticus med* cluster also lacks the expected amidinohydrolase [8]. We therefore sought to locate the "missing" amidinohydrolase by BLAST analysis [15] of our near-complete genome sequence of *S. mediocidicus* using as a probe the protein sequence of a putative amidinohydrolase (Orf32) from the biosynthetic gene cluster of the linear polyene ECO-02301 (Scheme 1) [16]. The analysis returned two strong matches, Medi4948 (80% identity, 93% similarity) and Medi2865 (82% identity, 92% similarity), both provisionally annotated as agmatinases (the numbers quoted refer to the position of the respective orf in the genome sequence). The next best match was a further agmatinase Medi0234 (41% identity, 57% similarity). Each of these three genes was cloned and expressed in *Escherichia coli* as an *N*-terminally histidine-tagged protein as described in the Experimental section, and purified by chromatography on a Ni-NTA column. The putative amidinohydrolases Medi2865 and Medi0234 were wholly inactive when incubated with desulfoclethramycin (**1b**) purified from DSM4137 extracts, although Medi2865 did show metal-dependent amidinohydrolase activity against 4-guanidinobutyrate to yield 4-aminobutanoate. In contrast, recombinant Medi4948 upon brief incubation gave essentially complete conversion of purified **1b** into mediomycin B (**1d**, see later). The gene encoding this enzyme is located 670 kbp distant from the *med* gene cluster on the *S. mediocidicus* chromosome (Scheme 2), a rare but not unprecedented [17] example of an apparently essential gene being located far from the relevant gene cluster. Because the *S. mediocidicus* strain proved highly resistant to introduction of cloned DNA, we were unable to obtain formal proof that this gene is essential for mediomycin biosynthesis by mutating *medi4948* and seeing accumulation of **1a** and/or **1b**. Instead, we introduced the *medi4948* gene, cloned in expression vector pIB139 [18] into DSM4137. As shown in Figure 1B, in extracts of the culture pellet of this recombinant the normal products of this strain **1a** and **1b** were absent, and replaced by mediomycins **1c** and **1d**, fully consistent with the proposed function of the amidinohydrolase encoded by gene *medi4948*. In contrast, when an authentic amidinohydrolase from the *Streptomyces olivaceus* Tü4018 strain producing the macrocyclic polyene desertomycin [10] was similarly cloned and expressed in *S. malaysiensis* DSM4137, it had no effect on the production of **1a** and **1b** (Figure S6, Supporting Information File 1), implying that these amidinotransferases operating on linear and macrocyclic polyenes do not have overlapping substrate specificities. Sequence alignment of amidinohydrolase Medi4948 with authentic ureohydrolases in the Protein Data Bank (PDB) (Figure S2, Supporting Information File 1) revealed that it contains the sequence motifs xGGDH, DAHxD, and SxDxDxxDPxxxP (where x = any amino acid), which are conserved in this enzyme superfamily and are implicated in cation binding and catalysis [19–22].

**Figure 2:**
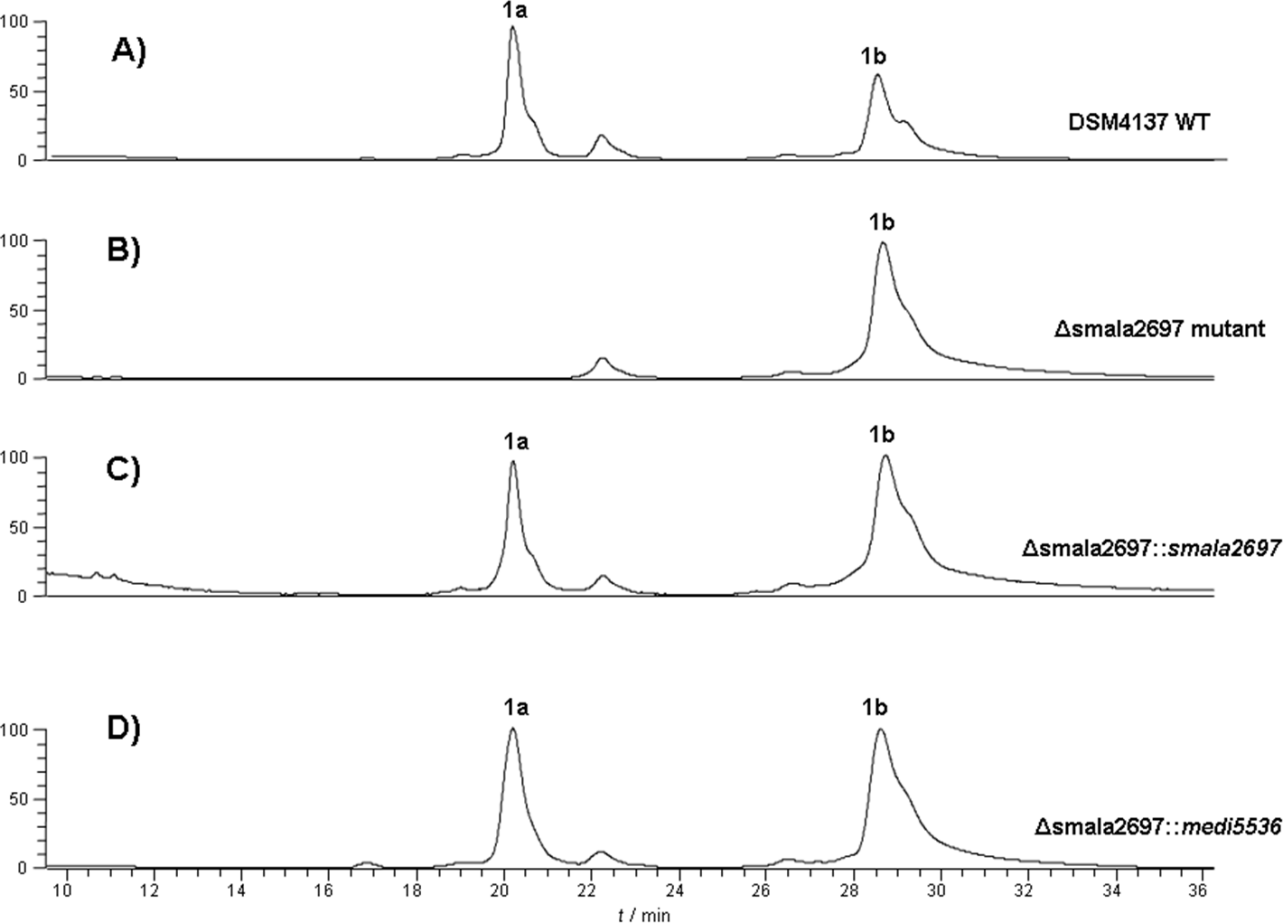
HPLC–UV–MS analysis of polyenes from DSM4137 wild type and mutants. A) LC–UV (360 nm) trace of mycelium methanol extract from DSM4137 wild type, showing the production of clethramycin (**1a**) and desulfoclethramycin (**1b**). B) LC–UV (360 nm) trace of mycelium methanol extract from the Δsmala2697 deletion mutant. In the mutant, production of **1a** was completely abolished. C) LC–UV (360 nm) trace of mycelium methanol extract from the Δsmala2697 deletion mutant complemented with *smala2697* from DSM4137. D) LC–UV (360 nm) trace of mycelium methanol extract from the Δsmala2697 deletion mutant complemented with the polyene cluster-associated *slf* gene (*medi5536*) from *S. mediocidicus*. Both complementations restored the production of sulfonated polyene **1a**.

Our finding that amidinohydrolase encoded by the *medi4948* gene acts on both desulfoclethramycin (**1b**) and clethramycin (**1a**) in vivo raised the question of whether there is a preferred or even obligatory order of events in the late-stage tailoring of the mediomycins in *S. mediocidicus*. In order to resolve this question we sought to characterise the putative sulfotransferase encoded by the polyene-cluster associated *slf* gene in both *S. malaysiensis* DSM4137 and *S. mediocidicus* (Table S4, Supporting Information File 1). The polyene cluster-associated *slf* gene in *S. malaysiensis* DSM4137 (*smala2697*) was specifically deleted in-frame as described in the Experimental section. The resulting mutant strain, under conditions where the wild type produces both **1a** and **1b**, only produced **1b** (Figure 2B) showing that the *slf* gene is uniquely responsible for the conversion of desulfoclethramycin into clethramycin. Complementation of the mutant was carried out with either a wild type copy of *slf* from DSM4137 (*smala2697*, or its counterpart from *S. mediocidicus* (*medi5536*). In each case, co-production of **1a** was fully restored (Figure 2C and 2D).

**Scheme 3:**
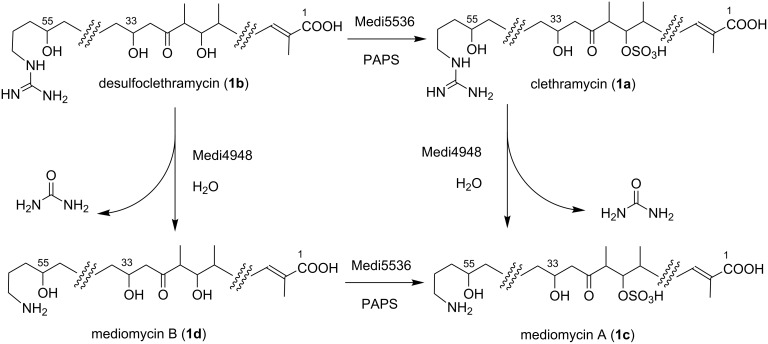
The parallel pathways for the biosynthesis of mediomycin A.

These in vivo results together establish that Medi4948 is capable of deprotection of both **1a** and **1b** and that the sulfotransferase SMALA_2697 (or Medi5536) is responsible for converting **1b** into **1a,** as indicated in Scheme 3.

**Figure 3:**
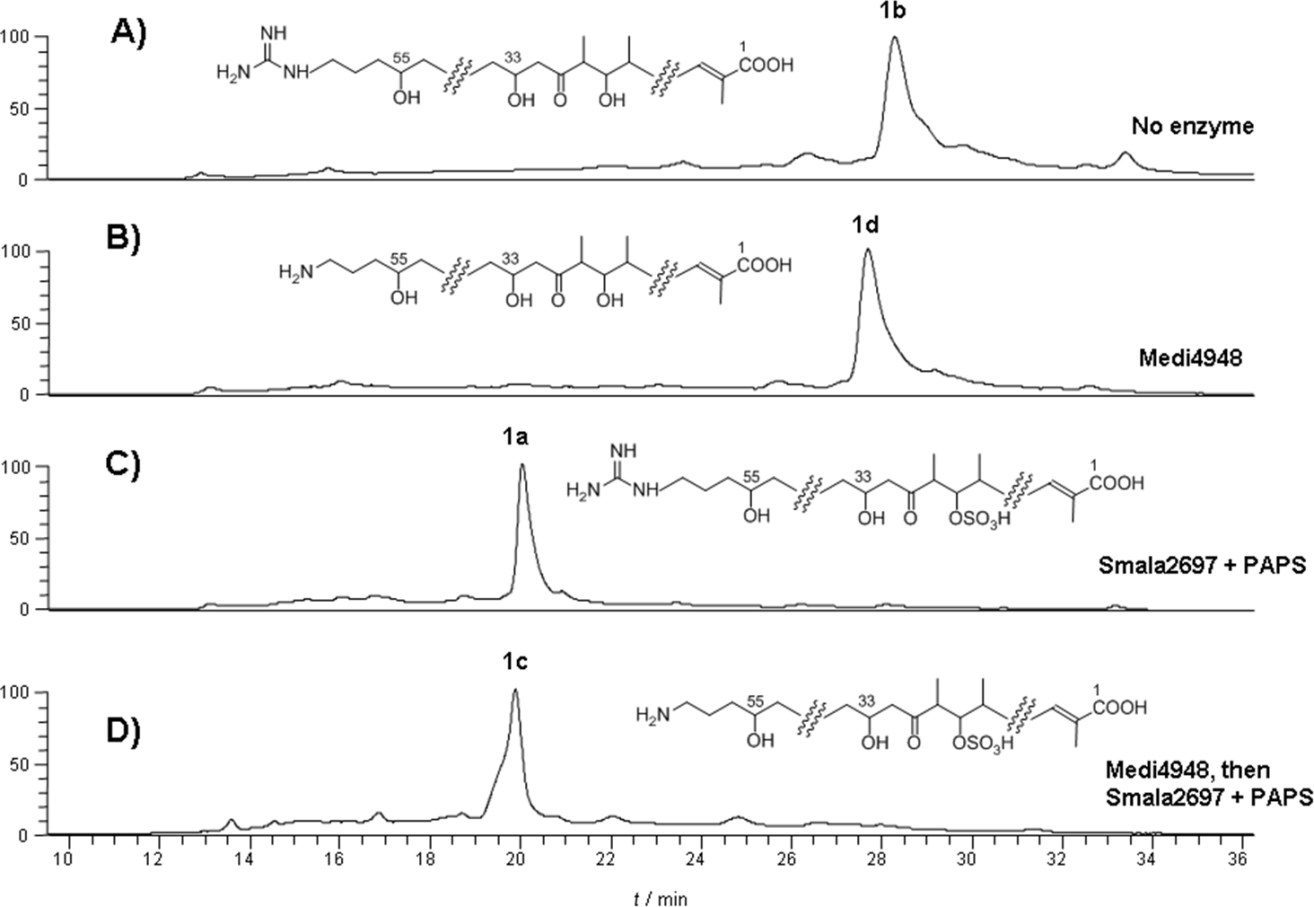
HPLC–UV–MS analysis of in vitro assays with amidinohydrolase Medi4948 and sulfotransferase Slf from *S. malaysiensis* DSM4137 (SMALA_2697). A) LC–UV (360 nm) trace of control assay, where purified **1b** at *m/z* 1138.8 ([M + H]^+^) was incubated without enzyme. B) LC–UV (360 nm) trace of **1b** incubated with amidinohydrolase Medi4948, showing complete convertion of desulfoclethramycin (**1b**) to its amino form mediomycin B (**1d**) at *m/z* 1096.8 ([M + H]^+^). C) LC–UV (360 nm) trace of **1b** incubated with sulfotransferase SMALA_2697 and PAPS, showing complete convertion of desulfoclethramycin (**1b**) to its sulfonated form clethramycin (**1a**) at *m/z* 1240.8 ([M + Na]^+^). D) LC–UV (360 nm) trace of **1b** incubated with amidinohydrolase Medi4948 to generate mediomycin B (**1d**) in situ, followed by addition of sulfotransferase SMALA_2697 and PAPS to convert mediomycin B (**1d**) to mediomycin A (**1c**) at *m/z* 1198.8 ([M + Na]^+^).

What remained unclear was whether the sulfotransferase is capable of acting on both **1b** and **1d**, which would mean that there are two independent routes to forming mediomycin A. We first confirmed that when purified recombinant Slf from DSM4137 (SMALA_2697) was incubated with **1b**, in the presence of the cofactor 3′-phosphoadenosine-5′-phosphosulfate (PAPS), **1a** was formed (Figure 3C). Then, **1b** was pre-incubated with amidinohydrolase Medi4948, to convert it fully into mediomycin B **1d**, as monitored by HPLC–MS (Figure 3B). Then, Slf from DSM4137 (SMALA_2697) was added to the reaction mixture together with PAPS. As shown in Figure 3D, **1d** was efficiently converted into **1c** under these conditions. Therefore parallel pathways do exist (Scheme 3) for the formation of mediomycin A (**1c**) from desulfoclethramycin (**1b**), which is the full-length initial product of the assembly-line PKS in both clethramycin and mediomycin biosynthesis. The sulfotransferases from the *cle* and *med* clusters appear essentially identical in their catalytic activity.

The co-location of the *slf* gene with the gigantic 27-extension module PKS of clethramycin or mediomycin suggested that the Slf protein sequence might be a useful probe to uncover further examples of strains producing these or closely-related compounds. BLAST analysis of public databases using the protein sequence of the Slf of DSM4137 as a probe sequence uncovered multiple candidate Slf sequences (see sequence alignment in Figure S3, Supporting Information File 1) all of which bear the conserved sequence motifs for binding of the sulfo-donor PAPS. Some, but by no means all, of the genes for these Slf sequences are indeed co-located with giant PKS genes (these are coloured green) in the phylogenetic tree of Slf sequences presented in Figure S3 (Supporting Information File 1). This analysis shows that *slf* genes associated with remarkable biosynthetic gene clusters harbouring 27-module PKS genes are distributed quite widely among *Streptomyces* strains. Because the mediomycin amidinohydrolase gene is not located anywhere near the *med* biosynthetic gene cluster, it is presently not possible to distinguish with confidence, on sequence comparisons alone, between clethramycin and mediomycin clusters.

## Conclusion

The accelerating speed and ever-decreasing cost of sequencing of microbial genomes has spurred our interest in a genome-led approach to the uncovering of novel enzymology in the biosynthetic pathways to antibiotic natural products [23–26]. Here, we have used in-house whole-genome sequencing to characterise the closely-related gene clusters to the sulfated antifungal linear polyenes clethramycin and mediomycin A. Further, it has enabled a successful search for the key amidinohydrolase enzyme required for late-stage deprotection of clethramycins to mediomycins, the gene for which is found 670 kbp distant from the *med* gene cluster. We have also shown that the amidinohydrolase can act either before or after the specific sulfonation step. The sequence of the sulfotransferase is a useful probe to uncover related gene clusters in public sequence databases.

Sulfonation remains a rare and relatively poorly understood modification in natural product biosynthesis [27–30]. As our understanding of their specificity improves, sulfotransferases have been deployed to increase the structural diversity of several classes of natural products [31–33]. The *slf* genes of polyene biosynthesis reported here represent an additional starting point for such natural product diversification. Sulfonation has been suggested as a novel approach to block the development of antibiotic resistance [33–34] while the discovery of the sulfated metabolite FR901379 was a critical breakthrough in the successful clinical development of micafungin to combat systemic fungal infections [12].

## Supporting Information

File 1 Experimental part and additional Figures and Schemes.
