# Supplementary material for: Sulfation and amidinohydrolysis in the biosynthesis of giant linear polyenes
Source: Beilstein J Org Chem. 2017 Nov 13;13:2408–15. doi: 10.3762/bjoc.13.238 (PMC5704753; doi:10.3762/bjoc.13.238)
Supplement: File 1 — Experimental part and additional Figures and Schemes. [file Beilstein_J_Org_Chem-13-2408-s001.pdf]

Supporting Information

for

Sulfation and amidinohydrolysis in the biosynthesis of giant linear polyenes

Hui Hong, Markiyan Samborskyy, Katsiaryna Usachova, Katharina Schnatz, Peter F. Leadlay*

Address: Department of Biochemistry, University of Cambridge, Cambridge CB2 1GA, U.K.

Email: Peter Leadlay* - pfl10@cam.ac.uk
*Corresponding author

Experimental part and additional Figures and Schemes

1. Supplementary methods

1.1. Bacterial strains and culture conditions

Streptomyces malaysiensis DSM4137 was obtained from the Leibnitz Institute - DSMZ. The strain was originally deposited in the collection in connection with a Hoechst patent application, so it is not listed in the DSMZ online catalogue, but it can be obtained by specific request via the European Patent Office quoting EP 0360130 A2. The strain has been referred to in previous publications from this laboratory as *Streptomyces violaceusniger* because its 16S rRNA sequence places it within the *S. violaceusniger* clade; and on the basis of its recently-obtained whole genome sequence this has been confirmed, and the strain can now be identified as a subspecies of *Streptomyces malaysiensis* (M.S., H.H. and P.F.L., *ms* in preparation). *Streptomyces mediocidicus* ATCC23936 was obtained from the American Type Culture Collection through LGC Standards (Teddington, Middlesex, U.K.). DSM4137 wild type and its mutants and *S. mediocidicus* were maintained on SFM agar (2% soya flour (Arkasoy), 2% D-mannitol, 2% agar) at 30 °C. For clethramycin, desulfoclethramycin,

mediomycin A and mediomycin B production, strains of DSM4137 and *S. mediocidicus* were cultured on fermentation liquid medium TSBY (3% TSB (Tryptic Soy Broth), 10.3% sucrose, 0.5% yeast extract) at 30 °C and 200 rpm in a rotary incubator and harvested after 2–3 days. *E. coli* strains were grown in Luria-Bertani (LB) broth (10% tryptone, 5% yeast extract, 10% NaCl) or agar (10% tryptone, 5% yeast extract, 10% NaCl, 2% agar) at 37 ^{o}C with appropriate antibiotic selection (kanamycin, at 50 µg mL^{-1}).

1.2. Materials, DNA isolation and manipulation

Bacterial strains, plasmids and oligonucleotides (Eurofins, Sigma) used in this work are summarised in Tables S1, S2 and S3, respectively. Restriction endonucleases were purchased from New England Biolabs (NEB). T4 DNA ligase and alkaline phosphatase were purchased from Fermentas. All chemicals were from Sigma-Aldrich. Liquid cultures for isolation of genomic DNA were grown in tryptone soya broth (Difco). DNA isolation and manipulation in *Streptomyces*, and *E. coli* were carried out using standard protocols [1,2]. PCR amplifications were carried out using Phusion® High-Fidelity DNA Polymerase (NEB). *E. coli* BL21(DE3) (Novagen) was used for protein expression.

1.3. Metabolite analysis and desulfoclethramycin isolation

For small-scale analysis, DSM4137 and *S. mediocidicus* were grown in liquid TSBY medium for 2-3 days. 1 mL samples of culture broth were centrifuged at 20,000*g* for 15 min. The mycelia pellets were then extracted with 1 mL of methanol at 60 °C for 2 hours. The mixture was spun down and the clear methanol extract was evaporated to dryness and dissolved in 200 µL of methanol. 10 µL of the extract was analyzed by LC–UV–MS. LC–UV–MS analyses were performed on a HPLC (Agilent Technologies 1200 series) coupled to a Thermo Fisher LTQ mass spectrometer fitted with an electrospray ionization (ESI) source. The methanol extracts were loaded onto a Prodigy 5µ C18 column (4.6 × 250 mm, Phenomenex), and the samples were eluted using MQ containing 5 mM ammonium acetate (A) and acetonitrile (B) at a flow rate of 0.7 mL min^{-1}. The elution gradient for both extracts was 5% to 35% B over 10 min, 35% to 65% B over 30 min. The elution was monitored at 360 nm as well as diode array detector (DAD). The mass spectrometer was run in positive ionization mode, scanning from *m/z* 200 to 2000 in full scan mode. MS/MS analysis were performed on $[M + H]^+$ ions with a normalized collision energy of 30%. High-resolution mass analysis was carried out on a Thermo Fisher Orbitrap mass spectrometer with resolution set up at 60 K.

For desulfoclethramycin production and isolation, four 1 L Erlenmeyer flasks with spirals, containing 200 ml TSBY medium, were inoculated with 5 mL 2-day TSBY seed culture of *S. malaysiensis* DSM4137 and incubated at 30 °C, 200 rpm. After 2 days, the broth was centrifuged at 9,500 rpm for 30 min. The pellet was resuspended in methanol and incubated at 60 °C for 2 h. The suspension was centrifuged at 2500*g* for 10 min at room temperature and the supernatant, which showed a significant yellow colour, was transferred to a round bottom flask. The solvent was evaporated and the water was removed by lyophilisation. The residue was dissolved in MeOH and purified by preparative HPLC (Agilent 1200) fitted with a Luna C18 column (100Å, 21.20 × 250 mm, Phenomenex). Compounds were eluted with 5 mM ammonium acetate (A) and MeOH (B) with a linear gradient of 5% to 60% B over 10 min, 60% B to 100% B over 20 min at a flow rate of 20 mL/min. Fractions were collected, and checked by MS analysis. Fractions containing desulfoclethramycin were combined. After removing methanol under reduced pressure, samples were lyophilized and kept at −20 °C before use.

1.4. Sulfotransferase gene knock-out in *S. malaysiensis* DSM4137

The knock-out of the sulfotransferase gene *smala2697* in *S. malaysiensis* DSM4137 was performed by introducing an in-frame deletion. The construction of the deletion plasmid pYH7-smala2697 was achieved by i) PCR amplification of around 2 kbp DNA fragments upstream and downstream of *smala2697*, using pairs of primers smala2697-L1/L2 and smala2697-R1/R2, respectively, from genomic DNA of *S. malaysiensis* DSM4137; ii) *NdeI* restriction digestion of the cloning vector pYH7, followed by treatment with antarctic phosphatase AnP, and agarose gel purification; iii) ligation of the two fragments and the digested pYH7 plasmid by the isothermal assembly method as described previously [3], with a 50 °C for 60 min incubation step; iv) transformation of pYH7-*slfV* in *E. coli* DH10B; v) plasmid isolation, and PCR and sequencing confirmation of the inserted deletion fragment, using primers smala2697-CP1, smala2697-CP2, NdeI-L, and NdeI-R.

The pYH7-smala2697 construct was then introduced into *S. malaysiensis* DSM4137 by intergeneric conjugation. Freshly grown *E. coli* ET12567-pUZ8002-pYH7-smala2697 cultures at A_{600} ~ 0.4–0.5 were thoroughly washed, to remove antibiotics, mixed with 2–3 days old *Streptomyces* mycelium, and plated on SFM agar. Following 20–22 h of incubation at 30 °C, plates were overlaid with nalidixic acid (25 μg mL^{-1}) and apramycin (5 μg mL^{-1}). Single *Streptomyces* colonies from these plates were streaked onto SFM agar containing 50 μg mL^{-1} apramycin, to confirm they had undergone antibiotic selection. Following further several rounds

of incubation in a non-selective TSBY medium, mutants were screened for Apr^S phenotype, by patching of single colonies onto both SFM agar and SFM agar containing apramycin (50 µg mL^{-1}). To identify the mutants in which a double cross-over event had occurred, their genomic DNA was amplified with the smala2697-CP1/CP2 primer pair, and the resulting DNA fragments of the correct length (0.7 kb) were verified by sequencing.

1.5. Complementation of amidinohydrolases into *S. malaysiensis* DSM4137

The amidinohydrolase *medi4948* was amplified by PCR, using as template genomic DNA of *S. mediocidicus,* and inserted into vector pIB139 via *Nde*I and *EcoR*V restriction sites to yield pIB139-medi4948.

The amidinohydrolase *amh_A828* was amplified by PCR, using as template genomic DNA of *Streptomyces olivaceus* Tü4018, and inserted into vector pIB139 via *Nde*I and *EcoR*V restriction sites to yield pIB139-amh828.

The construct was then introduced by conjugation into *S. malaysiensis* DSM4137. The donor strain was *E. coli* ET12567/pUZ8002, and conjugation was carried out on 20 mL of SFM plates. After incubating at 30 °C for 20 hours, exconjugants were selected with 50 µg mL^{-1} apramycin and 25 µg mL^{-1} nalidixic acid. Single colonies from this plate were transferred to a SFM plate containing 50 µg mL^{-1} apramycin to double check for antibiotic resistance. The patch from the confirmation plate was then inoculated into TSBY liquid culture containing 50 µg mL^{-1} apramycin for production of metabolites.

1.6. Complementation of the sulfotransferase deletion mutant of *S. malaysiensis* DSM4137 using cloned *slf* genes

The *in trans* complementation of the *S. malaysiensis* DSM4137 sulfotransferase deletion mutant Δsmala2697 was done using the native *smala2697*, as well as sulfotransferase *medi5536* from *S. mediocidicus* ATCC23936. Genes *smala2697* and *medi5536* were PCR amplified from genomic DNA, using primer pairs smala2697_com_F/R and medi5536_com_F/R, respectively. The cloning vector pIB139 was digested with *NdeI* and *Eco321* and gel purified. The *smala2697* and *medi5536* PCR fragments were ligated by the isothermal assembly method with the digested pIB139 plasmid, to yield plasmids pIB139-smala2697 and pIB139-medi5536, respectively. The latter plasmids were used to transform *E. coli* DH10B, the plasmids were isolated, and their identity confirmed by PCR and sequencing using primers

pIB-seqF and pIB-seqR. The constructs were then introduced by conjugation into the Δsmala2697 mutant. The conjugation procedure was as described in 1.4.

1.7. Protein expression and purification

The sulfotransferase gene *smala2697* was amplified by PCR, using genomic DNA of *S. malaysiensis* DSM4137 as template, and inserted into vector pET28a via *Nde*I and *Hind*III restriction sites to yield pET28a-smala2697.

The three amidinohydrolase genes *medi0234*, *medi2865* and *medi4948* were individually amplified by PCR, using genomic DNA of *S. mediocidicus*, and inserted into vector pET28a via *Nde*I and *Hin*dIII restriction sites to yield pET28a-medi0234, pET28amedi2865, and pET28a-medi4948. The identities of the plasmids were confirmed by DNA sequencing.

The plasmids were then used to transform *E. coli* BL21(DE3) for protein expression. A single colony was inoculated into 10 mL of LB medium containing 50 μg ml^{-1} kanamycin and grown overnight at 37 °C, 250 rpm. An aliquot (1 mL) was retained for preparation of a glycerol stock and the remaining culture was inoculated into 1 L LB medium containing 50 μg mL^{-1} kanamycin and incubated at 37 °C, 200 rpm until A_{600} reached 0.6 before addition of 400 μL of 1 M isopropyl-□-D-thiogalactopyranoside (IPTG) and incubation at 22 °C overnight to induce protein expression. Cells were harvested by centrifugation at 4000*g* for 10 min, resuspended in lysis buffer (20 mM Tris-HCl, pH 7.8, 0.5 M NaCl, 10 mM imidazole) and lysed by sonication. The total lysate was centrifuged at 14,000 x *g* for 40 min, and the supernatant was loaded onto a His-Bind column (1 mL bed volume), which had been precharged with nickel ions and equilibrated with lysis buffer. The column was washed with 10 column volumes of lysis buffer. Bound proteins were then eluted with a step gradient of increasing imidazole concentration (40, 80, 100, 150, 200, 250 and 500 mM in binding buffer). The protein solutions were concentrated, and further purified by gel filtration on an ÄKTA Explorer FPLC system fitted with a HiLoad 16/60 Superdex 200 Prep Grade column. The mobile phase contained 100 mM potassium phosphate, pH 7.4. Fractions containing protein of the expected size were pooled and concentrated using Amicon Ultra-4 concentrators (Millipore) fitted with either 10 kDa or 30 kDa filter. All purification steps were carried out at 4 °C. The purity of the protein was examined by 4–12% Bis-Tris Gel (Novex)

analysis and the concentration of the protein was measured by Bradford assay using bovine serum albumin as a standard.

1.8. In vitro activity assays

Amidinohydrolase activity with desulfoclethramycin as substrate

Each reaction mixture (50 µL) contained 10 µM purified candidate amidinohydrolase Medi2865 (or Medi4948, or Medi0234), 1 mM purified desulfoclethramycin in 100 mM potassium phosphate buffer pH 7.5. After incubation at 37 °C for 1.5 h, 10 µL of the reaction mixture was taken out, and mixed with 50 µL of methanol. The sample was clarified by centrifugation and then analyzed by HPLC–UV–MS.

Amidinohydrolase activity was also performed after pre-incubation of protein with various metal ions. Each reaction mixture (50 µL) contained 10 µM purified Medi2865 (or Medi4948, or Medi0234), 0.5 mM $MnCl_2$ (or $ZnCl_2$, or $MgCl_2$) in 50 mM Tris-HCl buffer pH 8.4. After incubation at 37 °C for 30 min, purified desulfoclethramycin was added to a final concentration of 1 mM, and the reaction was allowed to continue at 37 °C for 1 to 3 h. 10 □L of the reaction mixture was taken, mixed with 50 µL methanol, and after centrifugation analyzed by HPLC–UV–MS.

Amidinohydrolase activity with L-arginine (or 4-guanidinobutyric acid, 3guanidinopropionic acid, and 4-guanidinobutyramide) as substrate

Each reaction mixture (25 µL) contained 5 µM purified Medi2865 (or Medi4948, Medi0234), 0.5 mM $MnCl_2$ in 50 mM Tris-HCl buffer pH 9.0. After incubation at 37 °C for 30 min, L-arginine (or 4-guanidinobutyric acid, 3-guanidinopropionic acid, or 4guanidinobutyramide) was added to a final concentration of 1 mM, and the reaction was allowed to continue at 37 °C for 1 h. 12.5 µL of the reaction mixture was taken out, mixed with 10 µL of MQ and 3 µL of 1 M HCl, and analyzed by HPLC–MS with a Synergi 4□ Polar-RP column (4.6 × 250 mm, Phenomenex). Compounds were eluted using an isocratic gradient of 5% CH_3CN with 0.1% trifluoroacetic acid (TFA) for 10 min at a flow rate of 1.0 mL min^{-1}. The mass spectrometer was set to full scan mode (from *m/z* 100 to 1000), and MS^2 on $[M + H]^+$ ions at *m/z* 175.2, 145.2, 146.2 and 132.2 (for arginine, 4-guanidinobutyramide and 4-guanidinobutyric acid and 3-guanidinopropionic acid respectively) was performed with a normalized collision energy of 20%.

Sulfotransferase activity with desulfoclethramycin as substrate

Each reaction mixture (30 µL) contained 10 µM purified SMALA_2697, 1.5 mM 3′phosphoadenosine 5′-phosphosulfate (PAPS), 1 mM purified desulfoclethramycin in 100 mM potassium phosphate buffer pH 7.5. After incubation at 37 °C for 2 h, 10 µL of the reaction mixture was taken out, and mixed with 50 µL of methanol. After centrifugation, the sample was analyzed by HPLC–UV–MS.

Sulfotransferase activity with mediomycin B as substrate

Mediomycin B was generated in situ using desulfoclethramycin and amidinohydrolase Medi4948. Each reaction mixture (50 µL) contained 10 µM purified Medi4948, 1 mM purified desulfoclethramycin in 100 mM potassium phosphate buffer pH 7.5. After incubation at 37 °C for 1.5 h, 10 □L was taken out to check by LC–MS to make sure that desulfoclethramycin was fully converted to mediomycin B. Then purified SMALA_2697 and PAPS cofactor were added to the reaction mixture at a final concentration of 10 µM and 1.5 mM respectively, and the reaction was allowed to continue at 37 °C for 2 h. 10 µL of the reaction mixture was taken out, and mixed with 50 µL of methanol. After centrifugation, the sample was analyzed by HPLC–UV–MS.

2. Supplementary Scheme and Figures

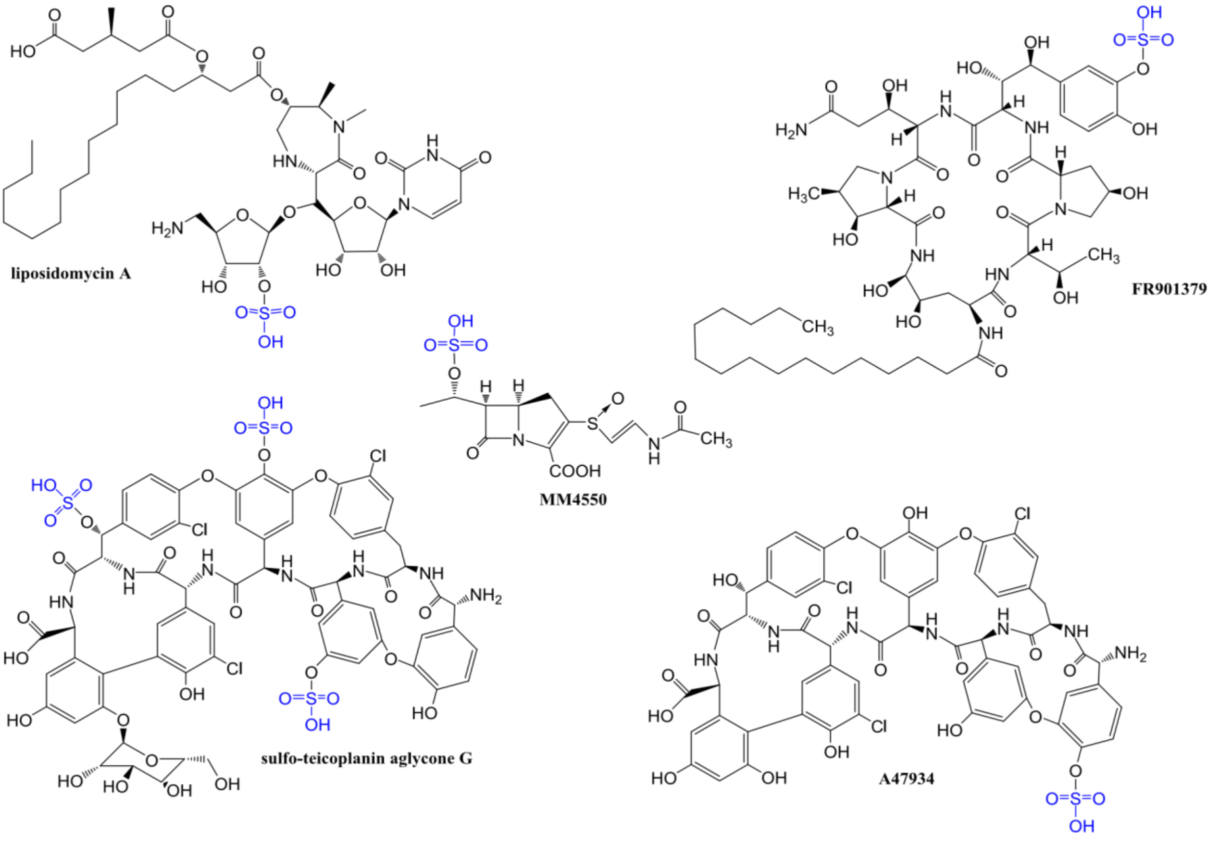

Scheme S1: Sulfonated natural products.

A)

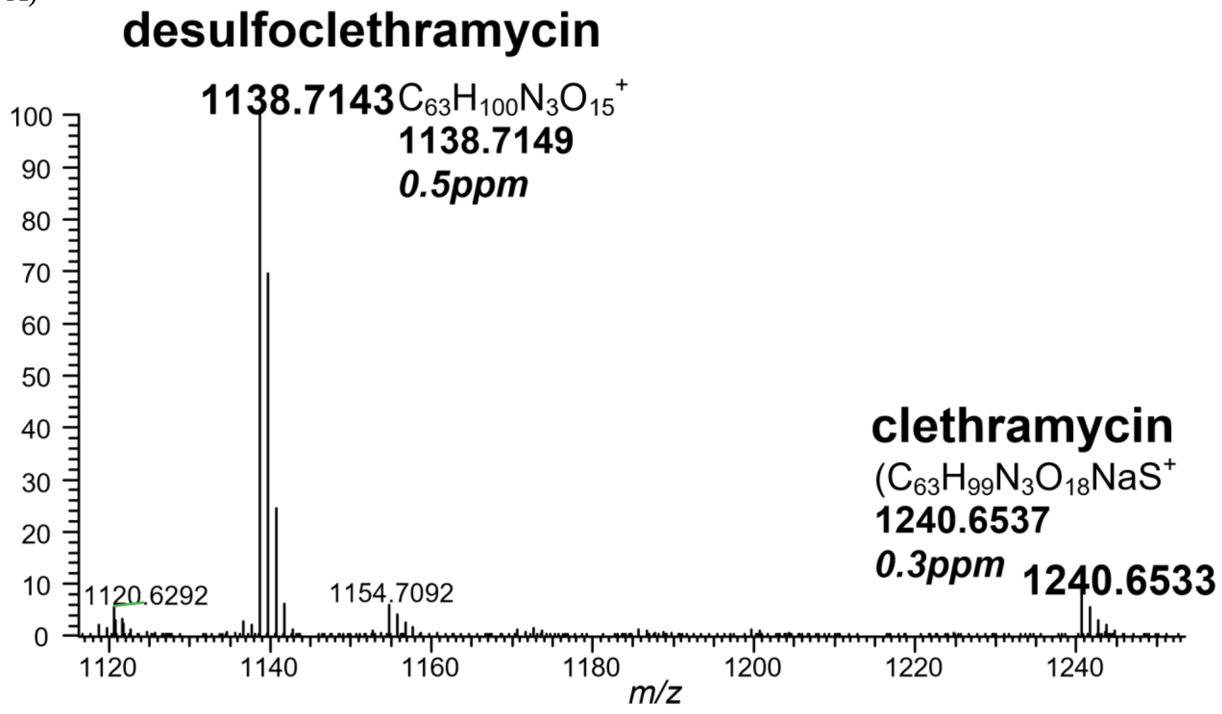
desulfoclethramycin
1138.7143
$C_{63}H_{100}N_3O_{15}^+$
1138.7149
0.5ppm
1120.6292
1154.7092
clethramycin
$(C_{63}H_{99}N_3O_{18}NaS^+$
1240.6537
0.3ppm
1240.6533
m/z

B)

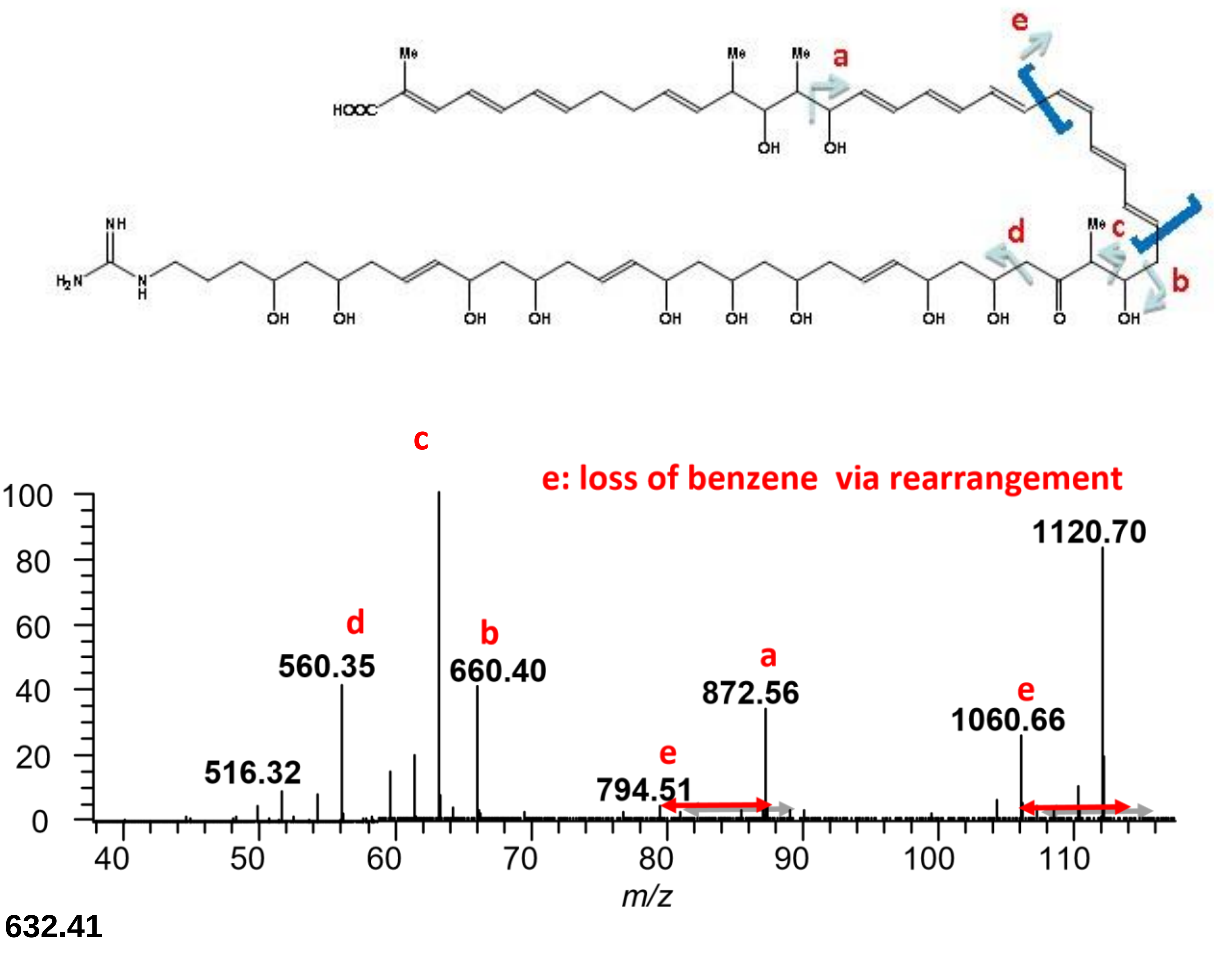
HOOC
Me
OH
a
b
c
d
e
NH
H_2N
H
O
e: loss of benzene via rearrangement
1120.70
560.35
660.40
872.56
1060.66
516.32
794.51
m/z
632.41

C)

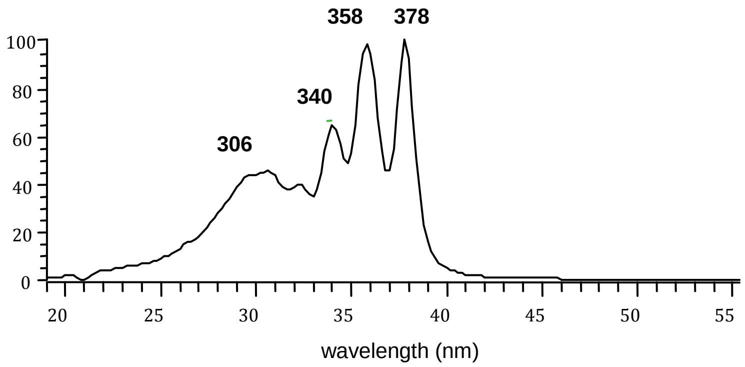

Figure S1: A) High-resolution MS (Orbitrap) analysis of clethramycin and desulfoclethramycin from *S. malaysiensis* DSM4137. B) High-resolution MS/MS analysis of desulfoclethramycin at *m/z* $[M+H]^+$: 1138.8. C) UV spectrum of desulfoclethramycin.

A)

```
                         10         20         30         40         50         60
Agm_CD       ---------- ---------- ---------- ---------- ---------- ----------
Agm_TV       ---------- ---------- ---------- ---------- ---------- ----------
ARG_BC       ---------- ---------- ---------- ---------- ---------- ----------
ARG_TT       ---------- ---------- ---------- ---------- ---------- ----------
Agm_DR       ---------- ---------- ---------- ---------- -------YGG IPTFARAPLV
AMH_A828     --MSETPESE AWRREVDRST FPRREPG--- -PI----DLR RYYVQPSYSG VPTFMGVPLA
AMH_A821     VKSEHTDDSA SWPYPIK--- QASRDPG--- -PL----NVH RNANQPAYVG IPTFMSLPIC
Medi0234     ---VTI---- --------PA TPGRPGGAAD APQDYDDAVH RADYRGVFGP QTSFLGLPQR
GbuA_PA      ---------- ---------- ---NLHQ--- -PLGG----- --NEMPRFGG IATMMRLPH-
GpuA_PA      ---------- ---------- -----PQ--- -PLDA----- --AEIPRFAG IPTFMRLPA-
Agm_BT       ---------- ---------- ---------- ---------- ---------- ----------
PAH_SC       ---------- ---------- ---------- ---------- ----SPRYAQ IPTFMRLPH-
SMALA_7636   --MTAN---- --------PP GPARPVP--- -PQET----- --ETTLHFTG PATFGRVPR-
AMH_SA       --MTAH---- --------PN GVTPPLP--- -PTET----- --DRTLHFAG PATFGRIPR-
SMALA_0333   ---------- ---------- -MTEPRG--- -PVDS----- --SRVPRFAG PATFARLPR-
Medi2865     ---MST---- --------TP APREPRG--- -PVDS----- --SRIPRYAG PATFARLPR-
Medi4948     ---MTF---- --------PN DKTPPVA--- -PEES----- --ERTLRFAG LPTFGRLPR-


                         70         80         90        100        110        120
Agm_CD       -----LNYEE SNLIVFGVGF DGTTSNRPGA RFASSSXRKE FYGLE-TY-- SP---FLDLD
Agm_TV       ---------- ----VFGIPF DNTSSYRRGS KYAPDSIRGA YVNLE-SY-- EY---SYGID
ARG_BC       ---------- --ISIIGVPM DLGQTRR-GV DMGPSAMRYA GVIERLER-- L---------
ARG_TT       ---------- --VAVVGVPM DLGANRR-GV DMGPSALRYA RLLEQLED-- L---------
Agm_DR       --QPDG-DWQ ADVAALGVPF DIALGFRPGA RFAPRALREA SLRSVPPFTG L----DGKTR
AMH_A828     LTQEDLRAGE VDVAVVGCPV DVSSGHR-GA AYGPRAIRAD ERYLYATPEG FVHSATRVNP
AMH_A821     LTPEDLRAGD VDVAVLGAPV DTSTGHR-GA AFGPRALRAD ERYLFNNTSD LVNASTRIKP
Medi0234     EQSPAG-YAG ADVVILGAPF DGTTSHRPGT RFGPQAIRRT DYLPHIPY-- RPHLGLGIDP
GbuA_PA      VQSPAE-LDA LDAAFVGVPL DIGTSLRSGT RFGPREIRAE SVMIR-PY-- NM-A-TGAAP
GpuA_PA      FTDPAA---- LQVGLIGVPW DGGTTNRAGA RHGPREVRNL SSLMR-KV-- HH-V-SRIAP
Agm_BT       ---------- --------PL DLATTFRSGA RLGPSAVRAA SVQLA-EL-- NP-YPWGFDP
PAH_SC       DPQPRG---- YDVVVIGAPY DGGTSYRPGA RFGPQAIRSE SGLIH-GV-- GI-D-RGPGT
SMALA_7636   LDQVDT---- ADIAVVGVPF DAGVSYRPGA RFGANAIREA SRQLR-PY-- NP-A-QDAYP
AMH_SA       IDQVEK---- TDIAVVGVPF DSGVTYRPGA RFGGNAIREA SRTLR-PY-- NP-A-QNVYP
SMALA_0333   LDEVAG---- ADVAVVGVPF DGGVSYRPGA RFGPAAVREA SRLLR-PY-- NP-G-LDVSP
```

```
Medi2865     LDEVGT---- ADVAVVGVPF DSGVSYRPGA RFGGNAIREA SRLLR-PY-- NP-A-QDASP
Medi4948     IEDVKE---- ANVAVVGVPF DSGVSYRPGA RFGGNAIREA SRMLR-PY-- NP-A-QDVYP

                    130        140        150        160        170        180
Agm_CD       LEDYNICDYG DLEISVGSTE ---------- -------QVL KEIYQETYKI VRDSKVPFXI
Agm_TV       LLASGXADLG DXEESED-VE ---------- -------YVI DTVESVVSAV XSDGKIPIXL
ARG_BC       --HYDIEDLG DIPIGKAERL HEQGDS--RL RNLKAVAEAN EKLAAAVDQV VQRGRFPLVL
ARG_TT       --GYTVEDLG DVPVSLARAS RRRGRGLAYL EEIRAAAL-- --VLKERLAA LPEGVFPIVL
Agm_DR       LQGVTFADAG DVILPSLEPQ ---------- -------LAH DRITEAARQV RGRCRVPVFL
AMH_A828     FNILKVVDYG DAAVDPFDIT ---------- -------RSM EPIRGLVREI AEVGARPVVL
AMH_A821     FDELTVVDYG DAAVDLWSIE ---------- -------NTE RTIGQVVSEV LDVGAVPLVM
Medi0234     FTELTVVDAG DVPTPPGETE ---------- -------RAH GLLERAVGEI VAAGAIPFTL
GbuA_PA      FDSLNVADIG DVAINTFNLL ---------- -------EAV RIIEQEYDRI LGHGILPLTL
GpuA_PA      YDLVRVGDLG DAPVNPIDLL ---------- -------DSL RRIEGFYRQV HAAGTLPLSV
Agm_BT       FDDLAVIDYG DCWFDAHHPL ---------- -------SIK PAIVEHARTI LQSDARMLTL
PAH_SC       FDLINCVDAG DINLTPFDMN ---------- -------IAI DTAQSHLSGL LKANAAFLMI
SMALA_7636   FHYVQVADAG DITANPHDID ---------- -------QAV QSVEAGTDAL LSTGARLMTL
AMH_SA       FHFSQVADAG DISANPFDLN ---------- -------DAV ETIEAAADDL ISSGARLMTL
SMALA_0333   FATQQVADAG DIAVNPFDIG ---------- -------EAI ETIQDAAGHL QADGARLVTI
Medi2865     FALAQVADAG DIAANPFNIN ---------- -------EAV ETIEAAADDL LGTGARMMTL
Medi4948     FHYSQVADAG DISANPFNIN ---------- -------EAV ETIEAAADGL LATDTRLMTL

                    190        200        210        220        230        240
Agm_CD       GGEHLVTLPA FKAVHEKYN- -DIYVIHFDA HTDLREEYNN SK-NSHATVI KRIWDI----
Agm_TV       GGEHSITVGA VRALPK---- -DVDLVIVDA HSDFRSSYXG NK-YNHACVT RRALDL----
ARG_BC       GGDHSIAIGT LAGVAKHYE- -RLGVIWYDA HGDVNTAETS PSGNIHGMPL AASLGFGHPA
ARG_TT       GGDHSLSMGS VAGAAR-GR- -RVGVVWVDA HADFNTPETS PSGNVHGMPL AVLSGLGHPR
Agm_DR       GGDHSVSYPL LRAFADVP-- -DLHVVQLDA HLDFTDTRND TK-WSNSSPF RRACEA----
AMH_A828     GGDHSLLWPS VGALSEVHGR GSIAVIHFDA HPDCHEELFG HR-ATHTTPI RRLIDE----
AMH_A821     GGDHSVMVPN VRALVEKYGA DKLAVVHFDA HPDCHEEIYG HT-KTHATTI WRLVNE----
Medi0234     GGDHSVAWPT MRGIAGRRGA GTFSVIHFDA HADIGDTSDF GSKYGHGTVM RRVLES----
GbuA_PA      GGDHTITLPI LRAIXKXXHG- -XVGLVHVDA HADVNDHMFG EX-IAHGTTF RRAVEE----
GpuA_PA      GGDHLVTLPI FRALGRE-R- -PLGMVHFDA HSDTNDRYFG DNPYTHGTPF RRAIEE----
Agm_BT       GGDHYITYPL LIAHAQKYG- KPLSLIHFDA HCDTWADDAP DS-LNHGTMF YKAVKD----
PAH_SC       GGDHSLTVAA LRAVAEQHG- -PLAVVHLDA HSDTNPAFYG GR-YHHGTPF RHGIDE----
SMALA_7636   GGDHTIALPI LRSVARRHG- -PVALLHFDA HLDTWDTHFG AQ-YTHGTPF RRAAEE----
AMH_SA       GGDHTIALPM LRAVAKKHG- -PLAVLHFDA HLDTWDDYFG QQ-YTHGMPF RRAVEE----
SMALA_0333   GGDHTIALPL LRAAARRHG- -PVAVLHFDA HLDTWDTYFG AE-HTHGTPF RRAVEE----
Medi2865     GGDHTIALPL LRSVAKKHG- -PVALLHFDA HLDTWDTYFG AE-YTHGTPF RRAVEE----
Medi4948     GGDHTIALPL LRSVAKKYG- -PVALLHFDA HLDTWDTYFG AE-YTHGTPF RRAVEE----

              *                                   *  **        ▲

                   250        260        270        280        290        300
***D*
Agm_CD       ---------- VGDNKIFQFG IRSGT---KE EFKFATEEKH TYX-----EI GGIDTFENIV
Agm_TV       ---------- LGEGRITSIG IRSVS---RE EFEDPDFRKV SFISSFDVKK NGIDKYIEEV
ARG_BC       LTQIGGYSPK IKPEHVVLIG VRSLD---EG EKKFIREKGI KIYTMHEVDR LGMTRVMEET
ARG_TT       LTEVF---RA VDPKDVVLVG VRSLD---PG EKRLLKEAGV RVYTMHEVDR LGVARIAEEV
Agm_DR       ---------L PNLVHITTVG LRGLR-FDPE AVAAARARGH TIIPMDDVTA DL-AGVLAQL
AMH_A828     ---------EM VPGPNVIQVG IRTISGPDDQ LFNWMRRAGM RSHFMAEIER IGFAAVIDKV
AMH_A821     ---------LG VPGHNIVQAG IRTPGSPDNQ LFHWMRKAGI HTHFMAEIER LGLPAVVDKV
Medi0234     ---------GT VPGNRFAQIG LRGYW-PGPR TLAWAAELGV RSVTMHELRS RGLDTCLDEV
GbuA_PA      ---------DL LDCDRVVQIG LRAQG-YTAE DFNWSRXQGF RVVQAEECWH XSLEPLMAEV
GpuA_PA      ---------GL LDPLRTVQIG IRGSV-YSPD DDAFARECGI RVIHMEEFVE LGVEATLAEA
Agm_BT       ---------GL IDPKASVQVG IRTWN----- ----DDYLGI NVLDAAWVHE HGARATLERI
PAH_SC       ---------KL IDPAAMVQIG IRGHN-PKPD SLDYARGHGV RVVTADEFGE LGVGGTADLI
SMALA_7636   ---------GL LDTSALSHVG TRGSL-YCKE DLDEDTKLGF GIVTAADVMR RGVDDVVRQL
AMH_SA       ---------GI LDTSALSHVG TRGPI-YGKK DLDDDEKLGF GIVTSADVMR RGVDEVAQQL
SMALA_0333   ---------GI VDTSALSHVG TRGPL-YGKE DLTEDEKLGF GIVTSADVYR RGADEVADQL
```

```
Medi2865      --------GI LDTSALSHVG TRGPL-YGKK DLTDDEKMGF GIVTSADVMR RGVDEIADQL
Medi4948      --------GI LDTSALSHVG TRGPL-YGKQ DLEEDEKLGF GIVTSADVMR RGVDEVIDQL


                     310        320        330        340        350        360
Agm_CD        NXL---NGKN IYLTIDLDVL DASVFPGTGT PEPGGVNYRE FQEIFKIIKN SNINIVGCDI
Agm_TV        D-R---KSRR VYISVDXDGI DPAYAPAVGT PEPFGLADTD VRR---LIER LSYKAVGFDI
ARG_BC        IAYLKERTDG VHLSLDLDGL DPSDAPGVGT PVIGGLTYRE SHLAMEMLA- EAQIITSAEF
ARG_TT        LKHLQG--LP LHVSLDADVL DPTLAPGVGT PVPGGLTYRE AHLLMEILA- ESGRVQSLDL
Agm_DR        -----PRGQN VYFSVDVDGF DPAVIPGTSS PEPDGLTYAQ GMKILAAAA- ANNTVVGLDL
AMH_A828      IEEARAVADH VYLSLDIDVL DPAFAPGTGT PEPAGLTTRE LFTALRRIA- HETNLVGMDV
AMH_A821      IAEASDGAEV VYVSLDIDVV DPAYAPGTGT PEPGGLSGRE ILTAFRRLC- HELPVVGMDV
Medi0234      LGQ--LGHGP TYLTIDIDVV DPGMAPGTGT PEPGGLTSRE LLDAVRTCA- QRTDLVGAEI
GbuA_PA       REX--VGGGP VYLSFDIDGI DPAWAPGTGT PEIGGLTTIQ AMEIIRGC-- QGLDLIGCDL
GpuA_PA       RRV--VGAGP TYVSFDVDVL DPAFAPGTGT PEIGGMTSLQ AQQLVRGL-- RGLDLVGADV
Agm_BT        ESI--VGGRP AYLTFDIDCL DPAFAPGTGT PVAGGLSSAQ ALAIVRGL-- GGVNLIGADV
PAH_SC        REK--VGQRP VYVSVDIDVV DPAFAPGTGT PAPGGLLSRE VLALLRCV-- GDLKPVGFDV
SMALA_7636    KER--IGTRP LYISVDVDVL DPAHAPGTGT PEAGGLTSRE LLEIVRGL-- SDCHVVSADV
AMH_SA        RER--VGDRP LYISIDIDVL DPAHAPGTGT PEAGGLTSRE LLEILRGL-- ADCHLVSADI
SMALA_0333    RQR--IGDRP LYISIDIDCL DPAHAPGTGT PEAGGLTSRE LLEILRGL-- AGCRLVGADV
Medi2865      RQR--VGDRP LYISIDIDVL DPAHAPGTGT PEAGGLTSRE LLEIIRGL-- SSCNLVSADL
Medi4948      RQR--IGDRP LYISVDIDVL DPAHAPGTGT PEAGGMTSRE LLEIIRGL-- SECRLVSADV
                                             ▲

                         **
                     370        380        390        400        410        420
Agm_CD        VELSPDYDT- TGVSTVIACK ILRE------ ---------- ---------- ----------
Agm_TV        VEFSPLYDN- GNTSXLAAK- ---------- ---------- ---------- ----------
ARG_BC        VEVNPILDE- RNKTASVA-- ---------- ---------- ---------- ----------
ARG_TT        VEVNPILDE- RNRTAEMLVG LALSLLG--- ---------- ---------- ----------
Agm_DR        VELAPNLDP- TGRSELLMAR LVMETLC--- ---------- ---------- ----------
AMH_A828      VEVAPHLDA- GYSTAMNARR AVFEALTGLA LNRIKISSKN YA-NPIVAGE VRFPLK----
AMH_A821      VEVAPHLDP- GYHTALLARR VILESISGLA MRKAGISTRD YR-HPVVSGE IPFAMPARRS
Medi0234      VELSPPYDGP GEITAFLANR VVLEVLSGMA WRRRVASSAG NGGVPS---- ----------
GbuA_PA       VEVSPPYDT- TGNTSLLGAN LLYEMLCVL- ---------- ---------- ----------
GpuA_PA       VEVSPPFDV- GGATALVGAT MMFELLCLLA ESAA------ ---------- ----------
Agm_BT        VEVAPAYDQ- SEITAIAAAH VACDLLCLWR QRKAG----- ---------- ----------
PAH_SC        MEVSPLYDH- GGITSILATE IGAE------ ---------- ---------- ----------
SMALA_7636    VEVAPAYDH- AEITCVAASH IAYELITLMS RQIVFFRW-V KAHEPS---- ----------
AMH_SA        VEVAPAYDH- ADITSVAASH AAYELISIMS KQIAPVRW-G ATQ------- ----------
SMALA_0333    VEVAPAYDH- AEITSVAASH VAYDLISLLA LQKKREKT-D E--------- ----------
```

```
Medi2865      VEVAPAYDH- AEITSVAASH AAYELTTIMS RQIAAARD-- ---------- ----------
Medi4948      VEVAPAYDH- ADITAVAASH VAYEMVSIMS KQMAPAYW-S KP-------- ----------
                ▲
```

B)

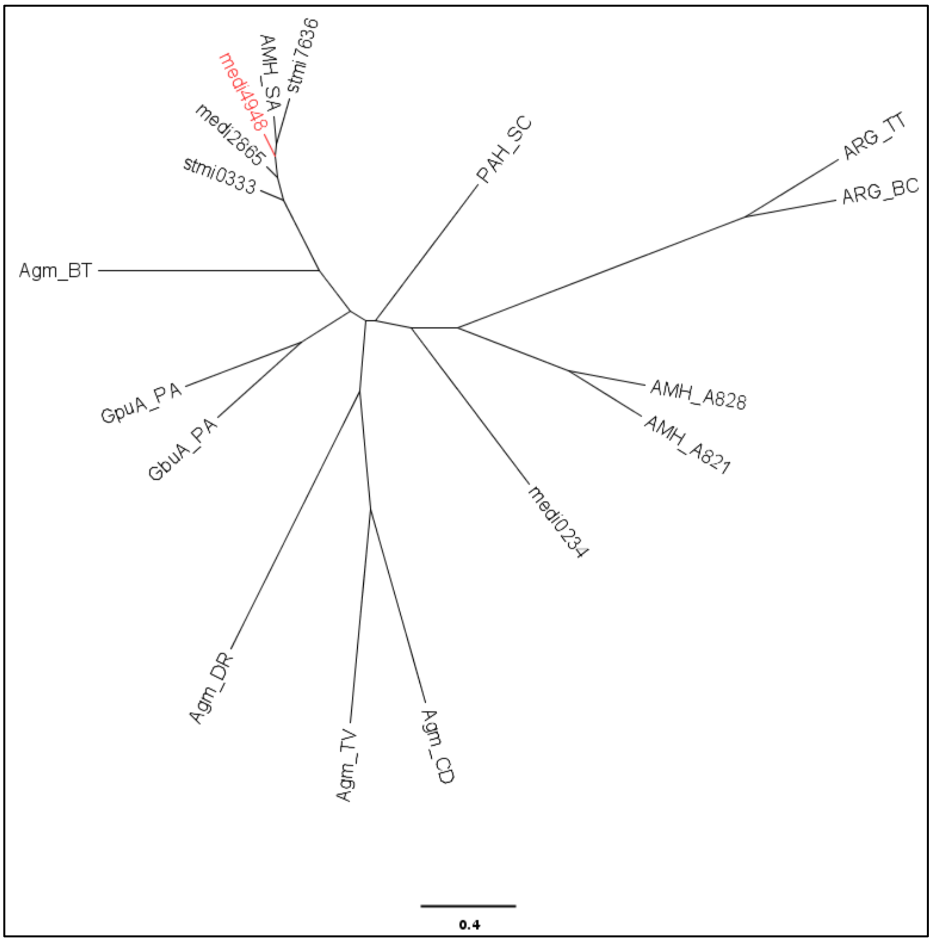

Figure S2: **A) Sequence alignment of ureohydrolases.** The sequences of seventeen ureohydrolases [AMH_A828: amidinohydrolase from *Streptomyces olivaceus* Tü4018 (A828); AMH_A828: amidinohydrolase from *Saccharomonospora azurea* (*caesia)* DSM 43044 (A821); GbuA_PA: guanidinobutyrase from *Pseudomonas aeruginosa*; GpuA_PA: guanidinopropionase from *Pseudomonas aeruginosa*; PAH_SC: proclavaminic acid amidino hydrolase (PAH) from *Streptomyces clavuligerus*; Agm_BT: agmatinase from *Burkholderia thailandensis*; Agm_DR: agmatinase from *Deinococcus radiodurans*; Agm_CD: agmatinase from *Clostridium difficile*; Agm_TV: agmatinase from *Thermoplasma. volcanium*; ARG_BC: arginase from *Bacillus caldovelox*; ARG_TT: arginase from *Thermus thermophilus*; AMH_SA: amidinohydrolase from *Streptomyces aizunensis*; medi0234, medi2865 and medi4948: amidinohydrolases from *Streptomyces mediocidicus*; SMALA_0333 and SMALA_7636: amidinohydrolases from *Streptomyces malaysiensis* DSM4137] are aligned using MultAlign. Three well-conserved

sequences (xGGDH, DAHxD, and SxDxDxxDPxxxP) in most of the ureohydrolases are indicated by black boxes. The metal binding sites are indicated with asterisks, guanidino ligands with black triangles. **B) Phylogenetic tree of amidinohydrolases medi4948 and homologues.** Construction of the phylogenetic tree was performed via the www.phylogeny.fr website, using default settings.

A)

```
                           ....|....| ....|....| ....|....| ....|....| ....|....| ....|....|
                                   10         20         30         40         50         60
SMALA_2697                 ---------- ---------- ---------- ---------- ---------- ----------
Sautol_AQA11921.1          ---------- ---------- ---------- ---------- ---------- ----------
Smelan_SEB92269.1          ---------- ---------- ---------- ---------- ---------- ----------
Smed_medi5536              ---------- ---------- ---------- ---------- ---------- ----------
Kmed9733_WP_035796292.1    ---------- ---------- ---------- ---------- ---------- ----------
Sblast_BAW35627.1          ---------- ---------- ---------- ---------- ---------- ----------
S.RTd22_NZ_CP015726.1      ---------- ---------- ---------- ---------- ---------- ----------
S.RK95-74_BAW35600.1       ---------- ---------- ---------- ---------- ---------- ----------
S.PRh5_EXU69913.1          ---------- ---------- ---------- ---------- ---------- ----------
Sviol_AEM87304.1           ---------- ---------- ---------- ---------- ---------- ----------
Srapa_AGP57770.1           ---------- ---------- ---------- ---------- ---------- ----------
Siran_CDR09769.1           ---------- ---------- ---------- ---------- ---------- ----------
Shygr_AQW50862.1           ---------- ---------- ---------- ---------- ---------- ----------
S.DSM7348_ORF0413          ---------- ---------- ---------- ---------- ---------- ----------
SMALA_0226                 ---------- ---------- ---------- ---------- ---------- ----------
Smed_medi1571              ---------- ---------- ---------- ---------- ---------- ----------
MtubStf1_PDB_2ZQ5_A        MTRRPDRKDV ATVDELHASA TKLVGLDDFG TDDDNYREAL GVLLDAYQGE AGLTVLGSKM
MtubStf3_CCP45048.1        MKALRSSSRL SRWREW--AA PLWVGCNFSA WMRLLIRNRF AVHHSRWHFA VLYTFLSMVN
MavStf9_PDB_2Z6V_A         MS---DFDNI TTADDVFKLA AQRTGLSEI- -DSDSWREGL ALIVDEVNTS PVFTPFGRQR
Sargent_AGU42411.1         ---------- ---------- ---------- ---------- ---------- ----------
Teg12_PDB_3MGC_A           ---------- ---------- ---------- ---------- ---------- ---------M
Teg13_ACJ60996.1           ---------- ---------- ---------- ---------- ---------- ----------
Teg14_PDB_3NIB_A           ---------- ---------- ---------- ---------- ---------- ---------M
Stoyoc_PDB_2OV8            ---------- ---------- ---------- ---------- ---------- ---------M
Actin_AGS77324.1           ---------- ---------- ---------- ---------- ---------- ----------

                           ....|....| ....|....| ....|....| ....|....| ....|....| ....|....|
                                   70         80         90        100        110        120
SMALA_2697                 --------VV NQK------- ---------- --LTFVVGTG RCGSTALSQV INIHPDVLSI
Sautol_AQA11921.1          --------MV NQK------- ---------- --LTFVVGTG RCGSTALSQV INIHPDVLSI
Smelan_SEB92269.1          --------MV NQK------- ---------- --LTFVVGTG RCGSTALSQV INIHPDVLSI
Smed_medi5536              --------VH TDK------- ---------- --LTFVVGTG RCGSTALSAV VNLHPDVLSL
Kmed9733_WP_035796292.1    --------MH TRK------- ---------- --LTFVVGTG RCGSTALSQV VNLHPDMLSV
Sblast_BAW35627.1          --------VH TDK------- ---------- --LTFVVGTG RCGSTALSAV VNLHPDVLSL
S.RTd22_NZ_CP015726.1      --------VV NQN------- ---------- --LTFIVGTG RCGSTAMSQV VNLHPDVLSI
S.RK95-74_BAW35600.1       --------ML NQN------- ---------- --LTFVVGTG RCGSTALSQV INLHPDVLSV
S.PRh5_EXU69913.1          --------MV NQK------- ---------- --LTFVVGTG RCGSTALSQI INIHPDVLSI
Sviol_AEM87304.1           --------MV NQK------- ---------- --LTFVVGTG RCGSTALSQV INIHPDVLSI
Srapa_AGP57770.1           --------MT VRP------- ---------- --LTFVVGTG RCGSTALSRI LRLHPDLLSV
Siran_CDR09769.1           --------MV NQK------- ---------- --LTFVVGTG RCGSTALSQI INIHPDVLSI
Shygr_AQW50862.1           --------MV NQK------- ---------- --LTFVVGTG RCGSTALSQV INIHPDVLSI
S.DSM7348_ORF0413          --------VV NQK------- ---------- --LTFVVGTG RCGSTALSQV INIHPDVLSI
SMALA_0226                 --------VS VRP------- ---------- --LTFVVGTG RCGSTALSRV LRLHPDLLSV
Smed_medi1571              ---------- MRK------- ---------- --LTFVVGTG RSGSSALSRV LNAHPGVLSL
MtubStf1_PDB_2ZQ5_A        NRFFLRGALV ARLLSQSAWK QYPEHVDVAI KRPIFVTGLV RTGTTALHRL LGADPAHQGL
MtubStf3_CCP45048.1        SCLGLWQKIV FGRRVAETVI ADP------- --PIFIVGHW RTGTTLLHEL LVVDDRHTGP
MavStf9_PDB_2Z6V_A         VLDDATNALG RRLQVHAYIQ DHPEVLDAPV ERPLIVLGMP RTGTTVISYL LDQDPARRSL
Sargent_AGU42411.1         ---------- -MP------- ---------- --LTVVLGTG RCGSTMLSDL VNQHPGALSL
Teg12_PDB_3MGC_A           ASMTGGQQMG RGS------- ---------M NGIRWIASYP KAGNTWVRCM LAAYITGKAP
Teg13_ACJ60996.1           ---------- ---------- ---------M NGIRWIASYP KAGNTWLRCM LAAYITGEAP
Teg14_PDB_3NIB_A           ASMTGGQQMG RGS------- ---------M NGIRWIASYP KAGNTWLRCM LAAYITGKAP
Stoyoc_PDB_2OV8            ---------- ---------- ---------- --MCWIASYP KAGGHWLRCM LTSYVTGEPV
Actin_AGS77324.1           ---------- ---------- ---------M TRISWIVSYP KSGNTWLRSM LTTYIGGAPA
```

```
                             ....|....| ....|....| ....|....| ....|....| ....|....| ....|....|
                                    130        140        150        160        170        180
SMALA_2697                   NELFASVPER E--MLDETAL SGPEFWGYLS RPNPITNSMI KNGASAPEFL YHRVPGRRFD
Sautol_AQA11921.1            NELFASVPER E--MLDETAL SGPEFWGYLS RPNPITNSMI KNGASAPEFL YHRVPGRRFD
Smelan_SEB92269.1            NELFASIPDT E--MLDEAPL SGPEFWGFLS RPNLITNSMI KNGATPPEFL YHKLPKRRFD
Smed_medi5536                NELFASVSDP A--VLSEEPL SGPEFWGFLT RPNRVSDNMI RNGAPPSEFL YNRHPEWRYS
Kmed9733_WP_035796292.1      NELFASVPGP Q--VLGDEPM SGTEFWGHLA RPNPVADTML RNGAPPPEFL YNRRPRGRYR
Sblast_BAW35627.1            NELFASVSDP A--VLTEEPL SGAEFWGFLT RPNRVSDSLI RNGAPPSEFL YNRHPEWRYS
S.RTd22_NZ_CP015726.1        SELFASIPDP E--LLSDTPL SGPEFWGYLC RPNPVTNSMI KNGATTPEFL YPRLPKRRYD
S.RK95-74_BAW35600.1         NELFASIPDS G--VLDETPL SGPEFWEYLS RPNAASDTLI KNGAIPPEFL YHRVPEGRFD
S.PRh5_EXU69913.1            NELFASIPDT E--VLDENPL SGSDFWGFLS RPNVVTNSMI KNGAIPPEFL YHRMPKRRFD
Sviol_AEM87304.1             NELFASIPDA E--MLDEAPL SGPEFWGYLS RPNVITNSMI RNGATPPEFL YHKLPKRRFD
Srapa_AGP57770.1             SELIASL-EP D--ALPGAPL TGAEFWRILA APRSFANRVI RDGIPLPEYR YPHVKGGRFS
Siran_CDR09769.1             NELFASIPDS E--ILDEAPL SGADFWGFLS RPNVVTNSMI KNGAIPPEFL YHRVPKRRFD
Shygr_AQW50862.1             NELFASIPDA E--MLDEAPL SGPEFWGYLS RPNVVTNSMI KNGATPPEFL YHRMPKRRFD
S.DSM7348_ORF0413            NELFASIPDA E--MLDEAPL SGPEFWGYLS RPNVVTNSMI KNGATPPEFL YHRMPKRRFD
SMALA_0226                   SELIASL-EP D--ALPEAPL TGAEFWRILA TPRSFANRVI RDGIPLPEYR YPHVEG-RFS
Smed_medi1571                NEFLASL-GS G--ALPEGVL TGEEFRRLLT RPNPVFETMN RSGMPLPEFL YVKRP-GRYA
MtubStf1_PDB_2ZQ5_A          HMWLAE--YP QPRPPRETWE SNP-LYRQLD ADFTQHHAEN PGYTGL-HFM AAYELEECWQ
MtubStf3_CCP45048.1          TGYECL--AP HHFLLTEWFA --P----YVE FLVSKHRAMD NMDLSL-HHP QEDEFVWCMQ
MavStf9_PDB_2Z6V_A           LHWQCV--HP IPPASTETLR TDPRCLALLD EQRKILDAVT RAKMPLPHWE DADGPTEDMF
Sargent_AGU42411.1           SEFFACL-DP G--VFPEGTL DGPAFWKLLG TPRLKPNVLM SRGVTVPEYR YP-IGSGRYA
Teg12_PDB_3MGC_A             QVWN----DI ---DA----- ----ESLTL- ------EAML RFGDLPP--- ----------
Teg13_ACJ60996.1             QVWK----DV ---YA----- ----ATPVL- ------EGML RFGDLPP--- ----------
Teg14_PDB_3NIB_A             QTWK----DM ---ET----- ----VSLEL- ------EGML HLGDMPP--- ----------
Stoyoc_PDB_2OV8              ETWP----GI ---QA----- ----GVPHL- ------EGLL RDGEAPS--- ----------
Actin_AGS77324.1             ESLRRL-QDL I--------- --PDIHPMLA E--------- ---------- ---------G

                             ....|....| ....|....| ....|....| ....|....| ....|....| ....|....|
                                    190        200        210        220        230        240
SMALA_2697                   AETTGIPAIS VMVLPHLTDD PDTLFDELES EVTSWPTRRP AEHWTALFTS LGARFGNPDA
Sautol_AQA11921.1            AETTGIPAIS VMVLPHLTDD PDTLFDELES EVTSWPTRRP AEHWTALFTS LGARFGNPDA
Smelan_SEB92269.1            AETTGIPAIS VMVLPHLTDD PDALFDELES EVTSWPTRRP ADHWTALFAF LGARFGNPGA
Smed_medi5536                AATTGIPAIS MMVLPHLTDD PDGLLDELEP EVNSWPTRPA PLQWQALFAT LAERFGAPGA
Kmed9733_WP_035796292.1      VETTGIPAVS MMVLPHLTDD PDGLLDALEP EVSAWPVRSP ARHWEAFFDA LAVRFGDPGA
Sblast_BAW35627.1            AATTGIPAIS MMVLPHLTDD PDGLLDELEP EVNSWPSRPA PLQWQALFAT LAARFGGPGA
S.RTd22_NZ_CP015726.1        AETTGIPAIS VMVLPHLTDD PDTLLDELEP VVTAWPTRAP ADHWRALFAD LAARFGGPGT
S.RK95-74_BAW35600.1         AQTTGIPAIS LMALPHLTDE PDALFDALEA EVTSWPTRAP ADHWRALFAT LGARFGDPGA
S.PRh5_EXU69913.1            AETTGIPAIS VMVLPHLTDD PDALFDELAA EVTTWPTRRP ADHWTALFAT LGARFGNPGA
Sviol_AEM87304.1             AETTGIPAIS VMVLPHLTDD PDALFDELES EVTSWPTRRP ADHWTALFAS LGARFGNPGA
Srapa_AGP57770.1             VPGGGIPAVC MMTLPHLTDD PDALFDALEP ELSRRPAAPV ADHYRALFGL LGERFGGR-A
Siran_CDR09769.1             AETTGIPAIS VMVLPHLTDD PDALFDELAA EVTTWPTRRP ADHWTALFAT LGARFGNTGA
Shygr_AQW50862.1             AETTGVPAIS VMVLPHLTDD PDALFDELES EVTSWPTRRP ADHWTALFVS LGARFGNPGA
S.DSM7348_ORF0413            AETTGIPAIS VMVLPHLTDD PDALFDELES EATSWPTRRP ADHWTALFAS LGARFGNPGA
SMALA_0226                   VAGGGIPAVC MMTLPHLTDD PDALFDALEP ELARRPAAPV ADHYRALFGL LGERFGRR-A
Smed_medi1571                TGTTGIPALS LMVLPHLTDD PDGLLDGIEA QVATWPARTA AAHHEALFDL LAARFGRT-A
MtubStf1_PDB_2ZQ5_A          LLRQSLHSVS YEALAHVPSY ADWLSRQDWT -----PSYCR HRRNLQLIGL NDA----EKR
MtubStf3_CCP45048.1          GLPSPYLTIA FPN--RPPQY EEYLDLEQVA -----PRELE IWKRTLFRFV QQVYFRRRKT
MavStf9_PDB_2Z6V_A           IHNQDFKGLS WDSFLPTDRY ARWLFDEADM -----SSTYE YQKRYLQVLQ STA----PGS
Sargent_AGU42411.1           AGE--VPAIS LMTLPPLTDD PDGLYDRIRD EVTGWPPAPV SGQYLRLFSW WASSCGRE-V
Teg12_PDB_3MGC_A             ---AEPME-P VLVKTHLKAD VPVL------ ---------- -----GLYG- ----------
Teg13_ACJ60996.1             ---AEPME-P VLVKTHLKAD VPVL------ ---------- -----GLYG- ----------
Teg14_PDB_3NIB_A             ---TEPTK-P VLVKTHLKAD VPVL------ ---------- -----GLYS- ----------
Stoyoc_PDB_2OV8              ---ADPDE-Q VLLATHFTAD RPVL------ ---------- -----RFYR- ----------
Actin_AGS77324.1             DVPAWDDGGS ALVKTHFLPD VRVL------ ---------- ---------- ----------
```

```
                                ....|....| ....|....| ....|....| ....|....| ....|....| ....|....|
                                       250        260        270        280        290        300
SMALA_2697                      VVERTGMSIG RVPEMHRAFP EARFVHLYRE GPDCAVSMSR HFSFRMIPLL WEMADHCGLE
Sautol_AQA11921.1               VVERTGMSIG RVPEMHRAFP EARFVHLYRE GPDCAVSMSR HFSFRMIPLL WEMADHCGLE
Smelan_SEB92269.1               VVERTGLSIG RVPEMHRAFP EACFVHLYRE GPDCAVSMSR HFSFRMIPLL WEMAMHLGLE
Smed_medi5536                   VVERSGLSLG RVPQLRALFP QARFVHLFRN GPDCALSMSR HIAFRMLPML WEMAQRCGLE
Kmed9733_WP_035796292.1         VVERSGLSLG RVPQLRRLFP HARFVHLYRD GPDCALSMSR HVGFRLLLLM WEMADRCGLA
Sblast_BAW35627.1               VVERSGLSLG RVPQLRALFP QARFVHLFRN GPDCALSMSR HIAFRMLPML WEMAQRCGLE
S.RTd22_NZ_CP015726.1           VVERTGLSIG RVPEMRRCFP EARFLHLYRE GPDCAVSMSR HYSFRMIPLL REMADHCGLD
S.RK95-74_BAW35600.1            VVERSGMSIG RVAEMHRAFP EARFVHLYRE GPDCAVSMSR HVSFRMLPLG WEMAIRCGLE
S.PRh5_EXU69913.1               VVERTGLSIG RVPEMHRAFP EARFVHLYRQ GPDCAVSMSR HFSFRMIPML WEMATHCGLE
Sviol_AEM87304.1                VVERTGLSIG RVPEMHRAFP EARFVHLYRE GPDCAVSMSR HFSFRMIPML WEMAMHLGLE
Srapa_AGP57770.1                VVERSGYSLR SVPRLREVFP EARFVHLHRD GADCALSMSR HPGFRLIQLM TERA------
Siran_CDR09769.1                VVERTGLSIG RVPEMHRAFP EAHFLHLYRQ GPDCAVSMSR HFSFRMIPML WEMATLCGLE
Shygr_AQW50862.1                VVERTGLSIG RVPEMHRAFP EARFVHLYRE GPDCAVSMSR HFSFRMIPML WEMAMHLGLE
S.DSM7348_ORF0413               VVERTGLSIG RVPEMHRAFP EARFVHLYRE GPDCAVSMSR HFSFRMIPML WEMAMHLGLE
SMALA_0226                      VVERSGYSLR SVPRLREVFP EARFVHLHRD GADCALSMSR HPGFRLIQLM TERA------
Smed_medi1571                   VVERSGYSVQ WVPRLRAAFP YARFVHLFRD GPDCALSMSR HVGYRTIFLF RRIQELTGVK
MtubStf1_PDB_2ZQ5_A             WVLKNPSHLF ALDALMATYP DALVVQTHRP VETIMASMCS L--------- AQHTTE-GWS
MtubStf3_CCP45048.1             VILKNPTHSF RIKVLLEVFP QAKFIHIVRD PYVVYPSTIH LHKALYRIHG LQQPTFDGLD
MavStf9_PDB_2Z6V_A              WSLKMPSHSV HIEALLKVFP DARLIWAHRD PYKATGSLCN L--------- WRLPQSLVMN
Sargent_AGU42411.1              VVERSGASLR FLPELLTHFP AARFVHVHRD GPDSAVSMSR HPLFRLGVLI GDMRAELGVD
Teg12_PDB_3MGC_A                -------EA- ---------- TAKVLYLVRN PRDMLLSSMR MASI------ ----SRDDVE
Teg13_ACJ60996.1                -------EA- ---------- TAKVLYLVRN PRDMLLSSMR MASI------ ----SRDDME
Teg14_PDB_3NIB_A                -------EA- ---------- TAKVLYLVRN PRDILLSAMR MTAI------ ----SRDDME
Stoyoc_PDB_2OV8                 -------ES- ---------- TAKVVCLIRN PRDAMLSLMR MKGI------ ----PPEDVE
Actin_AGS77324.1                ---RLYREVS R--------- --KAVYIVRN PRDVLLSSLR AMHI------ ----SHDDTA

                                ....|....| ....|....| ....|....| ....|....| ....|....| ....|....|
                                       310        320        330        340        350        360
SMALA_2697                      TPEHLTAEHV AQLPADLAVL LSD-RYDPAL VWDRPIPISA -FGALWSDTI VDGLEKL---
Sautol_AQA11921.1               TPEHLTAEHV AQLPPDLAVL LSD-RYDPAL VWDRPIPISA -FGALWSDTI VDGLEKL---
Smelan_SEB92269.1               TPQHLTPQHA AQLPPDLAPL LSD-RYDPAL VMERPIPLSA -FGTLWSETI VDGLRKL---
Smed_medi5536                   SPYELTPEHA AQLPPDLAPL LTE-HYDPAL VLDRPIPLAA -FGAMWSQWI VDGVRHL---
Kmed9733_WP_035796292.1         SPHELTAEHA ATLPADLAPL LGE-RFDPAL VLDRPMPLSV -FGGLWSHLV VDGVDRL---
Sblast_BAW35627.1               SPYELTPEHA AQLPPDLAPL LTE-HYDPAL VLDRPIPLAA -FGAMWSQWI VDGVRHL---
S.RTd22_NZ_CP015726.1           SPYQLTPAHA AQLPPDLAPL LAD-RYDPAL VTERHIPLAE -FGTLWSETI VDGLAKL---
S.RK95-74_BAW35600.1            TPFELTPEHA DQLPPDLAPL LRD-EWDPAL VMDRPIPLTA -FGGLWSGLI VDGLEKL---
S.PRh5_EXU69913.1               TPRHLTPQHA AQLPPDLAPL LSD-RYDPAL VWDRPIPIDA -FGTLWSETI VDGLRKL---
Sviol_AEM87304.1                TPQHLTPQHA AQLPPDLAPL LSD-RYDPAL VMERPIPLSA -FGTLWSETI VDGLRKL---
Srapa_AGP57770.1                -------TST EDLPAGLAAL LSDDEADLRP LYERSVPVAE -FGELWSSTI VEGLGHL---
Siran_CDR09769.1                TPRLLTPQHA AQLPPDLAPL LSD-RYDPAL VWDRPIPIGA -FGTLWSETI VDGLKKL---
Shygr_AQW50862.1                TPQQLTPQHA AQLPPDLAPL LSD-RYDPEL VMERPIPLSA -FGTLWSETI VDGLRKL---
S.DSM7348_ORF0413               TPQQLTPQHA AQLPPDLAPL LSD-RYDPAL VMERPIPLSA -FGTLWSETI VDGLRKL---
SMALA_0226                      -------ETT EDLPAGLDAL LSDDDADLRP LYQRSVPIAE -FGRLWSATI VEGLERL---
Smed_medi1571                   DFTELTEADV RALPPDLAGV LGE-RIDPAL VWDRELPVEG -FGALWSELV ARGAGHL---
MtubStf1_PDB_2ZQ5_A             T--------- --------KF VG-------- --------AQ -IGADAMDTW SRGLERFNAA
MtubStf3_CCP45048.1             D--------- --------KV VS-------- ---------- --------TY VDLYRKLDEG
MavStf9_PDB_2Z6V_A              T--------- --------EL LDQ------- --------TE -MGRLAMWQM RYHVDRPLRA
Sargent_AGU42411.1              PYRSPDPRHA ELLPERLRPF APD-SLDAAA LADTDIPLVR -FGDMWSRA- TGALRHL---
Teg12_PDB_3MGC_A                KSRDFARKFI ANEGLGWNAL GAG------- --------GG VGLGSWPENV RSWTESS---
Teg13_ACJ60996.1                SSRTFAREFI AIEGNSMMKL SPG------- --------AG IG--SWPENV RSWTESS---
Teg14_PDB_3NIB_A                SSRTFARDFI ANEGLRMRGR GGG------- --------AG LG--SWPENV RIWTESS---
Stoyoc_PDB_2OV8                 ACRKIAETFI ADEGFSSVRI WAG------- --------EG ----SWPENI RSWTDSV---
Actin_AGS77324.1                ECRRIAEGFI AH-----ESF FAD------- -------RGR IGIGSWTESL RMWTSTDI-V

                                ....|....| ....|....| ....|....| ....|....| ....|....| ....|....|
                                       370        380        390        400        410        420
SMALA_2697                      -DAVPAEQRT ALSYENLLEE PEKELIRLAE F----IGVEP -WRSWLDASV AHLD------
Sautol_AQA11921.1               -DAVPAEQRT ALSYENLLEE PEKELIRLAE F----IGVEP -WRSWLDASV AHLD------
Smelan_SEB92269.1               -DDVPAEQRT ALSYETLLEE PEKELIRLAE F----IGVEP -HRTWLDASV AHLD------
Smed_medi5536                   -EELPADIRT ALSYERLLEE PRKELTRLAE F----IGVEA -RPDWLDAAT ALLD------
Kmed9733_WP_035796292.1         -EEIPQSQRS TLRYEQLIAD PRGELKRLAD F----AGVEA -DPRWLTAGA ALLD------
Sblast_BAW35627.1               -EELPADIRT ALSYERLLEE PRKELTRLAE F----IGVEA -RPDWLDAAT ALLD------
S.RTd22_NZ_CP015726.1           -EEVPADLRT AMSFETLLDE PEKELVRLAE F----LGVEP -LPAWLDAAT ALLD------
S.RK95-74_BAW35600.1            -EAVPESQRT ALPYEDLLEE PEKELIRLAE F----IGVEP -HPAWLEESV AHLD------
S.PRh5_EXU69913.1               -DEVPAEQRT ALSYETLLEE PEKELIRLAE F----IGVEP -HRDWLDASI AHLD------
Sviol_AEM87304.1                -DDVPAEQRT ALSYETLLEE PEKELIRLAE F----IGVEA -HRTWLDASI AHLD------
Srapa_AGP57770.1                -SRLPAAIRM SLSYEGLLDA PERELTRLAH H----LGVEP -LPEWLAAGR ALLD------
Siran_CDR09769.1                -DEVPAEQRA ALSYETLLEE PEKELIRLAE F----IGVEP -HRDWLDASI AHLD------
Shygr_AQW50862.1                -DDVPAEQRT ALSYESLLEE PEKELIRLAE F----IGVEP -HRTWLDASI AHLD------
```

```
S.DSM7348_ORF0413         -DDVPAEQRT ALSYETLLEE PEKELIRLAE F----IGVEP -HRTWLDASI AHLD------
SMALA_0226                -SRLPADIRM SLSYEGLLDA PERELTRLAH H----VGVEP -LPEWLAAGR ALLD------
Smed_medi1571             -AEVPAPQRT ALAYEDLLDR PEEELSRLAR F----VGVEP -LPEWLDAGR ALLD------
MtubStf1_PDB_2ZQ5_A       RAKYDSAQFY DVDYHDLIAD PLGTVADIYR HFG--LTLSD --EARQAMTT VHAE------
MtubStf3_CCP45048.1       RELVDPTRFY ELRYEDLIGD PEGQLRRLYQ HLG--LGDFE CYLPRLRQYL ADHA------
MavStf9_PDB_2Z6V_A        RERIGDERFF HMYYHEMMRD PMDVMRRIYE WADEPLTAET --EARMRNWL AHHP------
Sargent_AGU42411.1        -AGLPPERLL HLSYDAVVAG PVPQLTRFGR F----VGLAE -PQRWAERVA GQVD------
Teg12_PDB_3MGC_A          SDRFPNADVL TMRYEDLKGD PVARFSEIVE FLD--LGGPV -DIEDIRRAV AASTLERMRE
Teg13_ACJ60996.1          RDRFPNADVL TMRYEDLRAD PVARFSEIVE FLD--LGGPV -DIEDIRRAV AASTLERMRE
Teg14_PDB_3NIB_A          RDRFPNADVL TMRYEDLKGD PVARFSEIVE FLD--LGDPV -DIEDIRRAV AACTLERMRE
Stoyoc_PDB_2OV8           HESFPNAAVL AVRYEDLRKD PEGELWKVVD FLE--LGGRD -GVAD---AV ANCTLERMRE
Actin_AGS77324.1          RDSFPDIDVL TVRYEDMRSD PAGKLTEIVE FLD--LGRPI -VEHDIQGAV EGSTLDRMRE

                          ....|....| ....|....| ....|....| ....|....| ....|....| ....|....|
                                 430        440        450        460        470        480
SMALA_2697                ---------- ---------- ---------- ---------G -GRPGAARKL PTEELTSLLE
Sautol_AQA11921.1         ---------- ---------- ---------- ---------G -GRPGAARKL PTEELTSLLE
Smelan_SEB92269.1         ---------- ---------- ---------- ---------G -GRPGAARKL PEAELTPLLE
Smed_medi5536             ---------- ---------- ---------- ---------G DGRCGSALTL PPAELDALRE
Kmed9733_WP_035796292.1   ---------- ---------- ---------- ---------G -GRCGAALNL PADELAALRR
Sblast_BAW35627.1         ---------- ---------- ---------- ---------G DGRCGSALAL PPAELDALRE
S.RTd22_NZ_CP015726.1     ---------- ---------- ---------- ---------S -GRRGNALKL PEEERAALLD
S.RK95-74_BAW35600.1      ---------- ---------- ---------- ---------S -GRPGAARKL PEAELTPLLE
S.PRh5_EXU69913.1         ---------- ---------- ---------- ---------G -GRPGAASKL AEEELTSLLE
Sviol_AEM87304.1          ---------- ---------- ---------- ---------G -GRPGAARKL PEAELTPLLE
Srapa_AGP57770.1          ---------- ---------- ---------- ---------G DRRGTAAATL PPAELTALRE
Siran_CDR09769.1          ---------- ---------- ---------- ---------G -GRPGAASKL AEAELTSLLE
Shygr_AQW50862.1          ---------- ---------- ---------- ---------G -GRPGAARKL PEAELTPLLE
S.DSM7348_ORF0413         ---------- ---------- ---------- ---------G -GRPGAARKL PEAELTPLLE
SMALA_0226                ---------- ---------- ---------- ---------G DRRGTAAATL PPAELAALRE
Smed_medi1571             ---------- ---------- ---------- ---------G -SRRGASRRL PPGRLAALRE
MtubStf1_PDB_2ZQ5_A       ---------- ------SQS- ---------- ---------- -GARAPKHSY SLADYGLTVE
MtubStf3_CCP45048.1       ---------- ------D--- ---------- ---------- ---------Y KTNSYQLTVE
MavStf9_PDB_2Z6V_A        ---------- ------QD-- ---------- ---------- ---RFALNAY RLDEYGLTVE
Sargent_AGU42411.1        ---------- ---------- ---------- ---------R -KRAGAAARL SARQAEELRL
Teg12_PDB_3MGC_A          LEKRSEQQGG GSPIRHGDAR MMKGGPGGAR PQ-----FVG EGRYDQSLSF LGEDIESDYQ
Teg13_ACJ60996.1          LEKRSQQQGG GT-------- ----APGGQE SRSSGVPFVG EGRYDQSLSF LGEDIESAYQ
Teg14_PDB_3NIB_A          LEKRSQQQGQ WAS------- -MTGGRGGEK HP-----FVG EGRYDQSLSF LGEDIESAYQ
Stoyoc_PDB_2OV8           MEERSKLLGL ETTGLMT--- -----RGGKQ LP-----FVG KGGQRKSLKF MGDDIEKAYA
Actin_AGS77324.1          MEKKDKVNRK PPPLSRWAA- --------AK NPAQQFPFIG EGRQGQSLAF MGEDIEDAFR
                          ....|....| ....|....| ....|....| ....|
                                 490        500        510
SMALA_2697                SCSPGTRALA VHQ------- ---------- ----
Sautol_AQA11921.1         SCSPGTRALA VHQ------- ---------- ----
Smelan_SEB92269.1         SCSPGTRALA AHQ------- ---------- ----
Smed_medi5536             SCTTGTEALA GQGL------ ---------- ----
Kmed9733_WP_035796292.1   SCEPGMRALA RHGC------ ---------- ----
Sblast_BAW35627.1         SCTAGNEALA GQGL------ ---------- ----
S.RTd22_NZ_CP015726.1     ACAAGTRALA THP------- ---------- ----
S.RK95-74_BAW35600.1      SCTPGMRALA AHP------- ---------- ----
S.PRh5_EXU69913.1         SCSPGTRALA AHQ------- ---------- ----
Sviol_AEM87304.1          SCSPGTRALA AHQ------- ---------- ----
Srapa_AGP57770.1          SCAPGTRALS LAHGG----- ---------- ----
Siran_CDR09769.1          SCSPGTRALA AHQ------- ---------- ----
Shygr_AQW50862.1          SCSPGTRALA AHQ------- ---------- ----
S.DSM7348_ORF0413         SCSPGTRALV AHQ------- ---------- ----
SMALA_0226                SCAPGARALS AVHGG----- ---------- ----
Smed_medi1571             SCEPGTRALE AYRQRCQVS- ---------- ----
MtubStf1_PDB_2ZQ5_A       ---MVKERFA GL-------- ---------- ----
MtubStf3_CCP45048.1       QRAIVDEHWG EIIDRYGYDR HTPEPARLRP AVGG
MavStf9_PDB_2Z6V_A        ---ALQPIFA EYLDTFDIEL EGRP------ ----
Sargent_AGU42411.1        ACAPGTRRLT ALLGGTTDAA P--------- ----
Teg12_PDB_3MGC_A          ELLHGDSGFA LYAKQYGYAG ---------- ----
Teg13_ACJ60996.1          ELLHGDSEFA HYAKQYGYAG ---------- ----
Teg14_PDB_3NIB_A          ELLHGDSEFA HYAKQYGYAG ---------- ----
Stoyoc_PDB_2OV8           DLLHGETDFA HYARLYGYAE ---------- ----
Actin_AGS77324.1          ERLRDGSEFA LLAKQFGYDE ---------- ----
```

B)

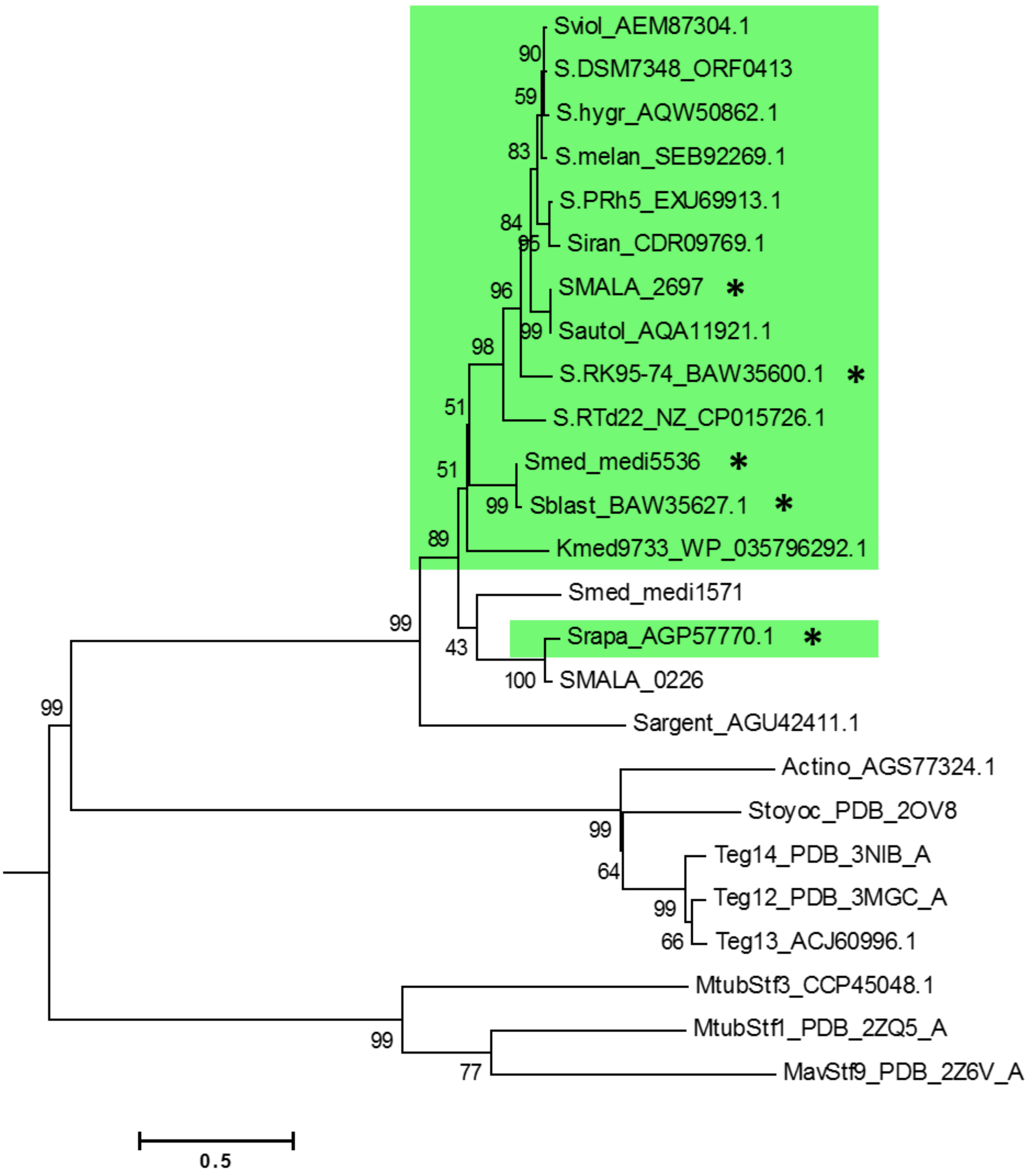

Figure S3: Phylogenetic analysis of sulfotransferase genes in actinobacteria. SMALA_2697 from *S. malaysiensis* DSM4137, Sautol from *S. autolyticus* CGMCC0516, Smelan from *S. melanosporafaciens* DSM40318, Smed_medi5536 from *S. mediocidicus* ATCC 23936, Kmed from *Kitasatospora mediocidica* KCTC9733, Sblast from *S. blastmyceticus* NBRC12747, S.RTd22 from *Streptomyces* sp. RTd22, S.PRh5 from *Streptomyces* sp. PRh5 CCTCC2013487, Sviol from *S. violaceusniger* Tü 4113, Srapa from

S. rapamycinicus NRRL5491, Siran from *S. iranensis* HM 35, Shygr from *S. hygroscopicus* XM201, S.DSM7348 from *Streptomyces* sp. DSM7348 – are sulfotransferases (Slf) associated with a clethramycin/mediomycin cluster. S.RK95-74 from *Streptomyces* sp. RK9574 has been reported to contain a neomediomycin cluster [4]. SMALA_0226 and Smed_medi1571 are additional Slf genes in *S. malaysiensis* DSM4137 and *S. mediocidicus*
ATCC 23936, respectively. Sargent is an Slf associated with the production of carbapenem MM4550 in *S. argenteolus* ATCC11009. MtubStf1 is an Slf involved in the production of sulfated trehalose glycolipids in *Mycobacterium tuberculosis* H37rv. MtubStf3 is an Slf involved in the production of sulfomenaquinone S881 in *M. tuberculosis* H37rv. MavStf9 is a *Mycobacterium avium* Slf of unknown function. Stoyoc is the StaL sulfotransferase in the teicoplanin A47934 biosynthetic pathway in *S. toyocaensis,* Actino is a tailoring Slf within the UK-68,597 glycopeptide biosynthetic cluster in *Actinoplanes* sp. ATCC. Teg12, Teg13, and Teg 14 are tailoring Slfs within the TEG cluster isolated from metagenomic DNA and predicted to produce a polysulfated teicoplanin-like glycopeptide. GenBank or PDB accession numbers are shown for each Slf sequence. **A) Sequence alignment of sulfotransferases.** The multiple alignment of amino acid sequences was performed using NCBI COBALT. Three well-conserved sequences of the 5′-PAP- and 3′-PAPS- binding motifs are indicated by a solid-line and dot-line boxes, respectively. **B) Phylogenetic tree of sulfotransferases.** Maximum-likelihood inference was performed in MEGA 6.06 on the WAG+I+G4+F substitution model with a bootstrap resampling of 1000 replicates. Nodes of the tree are labeled with bootstrap values, %. Slf genes associated with giant linear polyene biosynthetic gene clusters are shown shaded green. The Slf genes labelled with an asterisk denote known clethramycin, mediomycin or neomediomycin gene clusters.

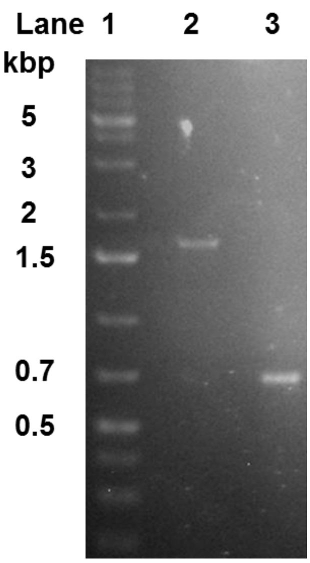

Figure S4*:* In-frame deletion of sulfotransferase gene *smala2697* in *Streptomyces malaysiensis* DSM4137. Lane 1: marker; Lane 2 and 3: PCR product from WT (1,642 bp) and Δsmala2697 (694 bp), respectively.

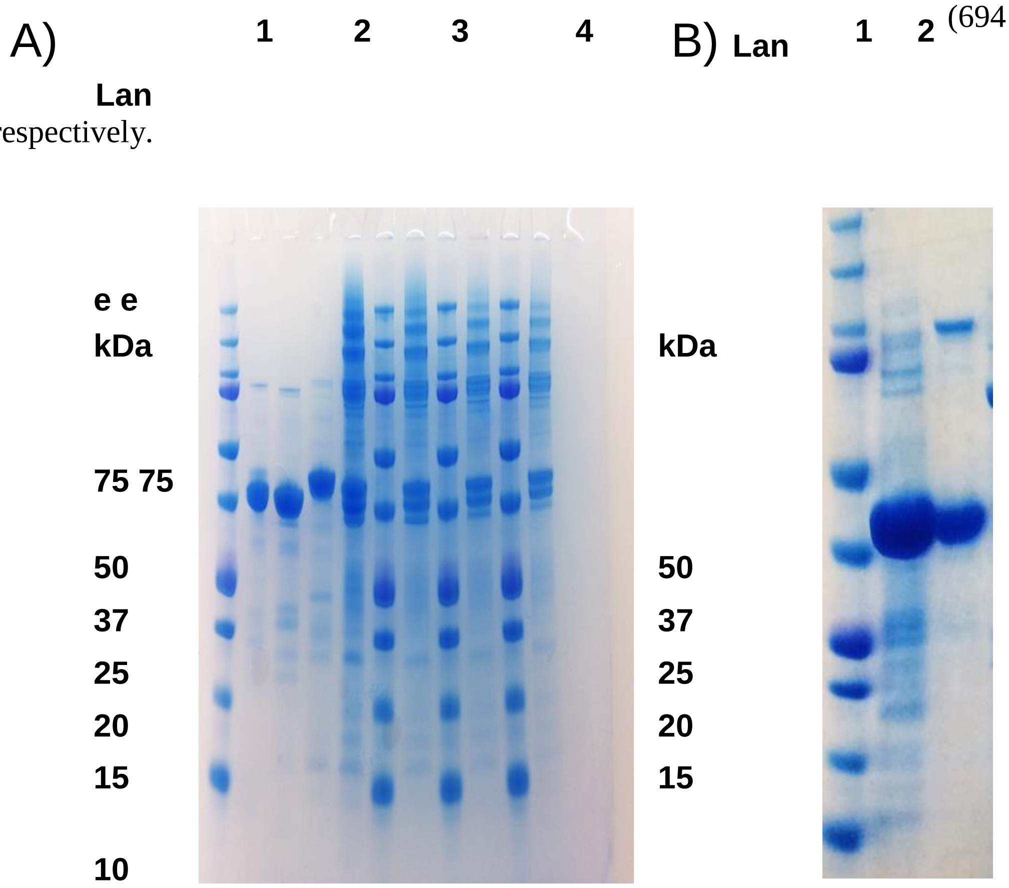

Figure S5: *4* - 12% Bis-Tris SDS-PAGE analysis of A) amidinohydrolases. Lane 1, protein standards; Lane 2, Medi4948; Lane 3, Medi2865; Lane 4, Medi0234. **B) Sulfotransferase SMALA_2697.** Lane 1, protein standards; Lane 2, SMALA_2697.

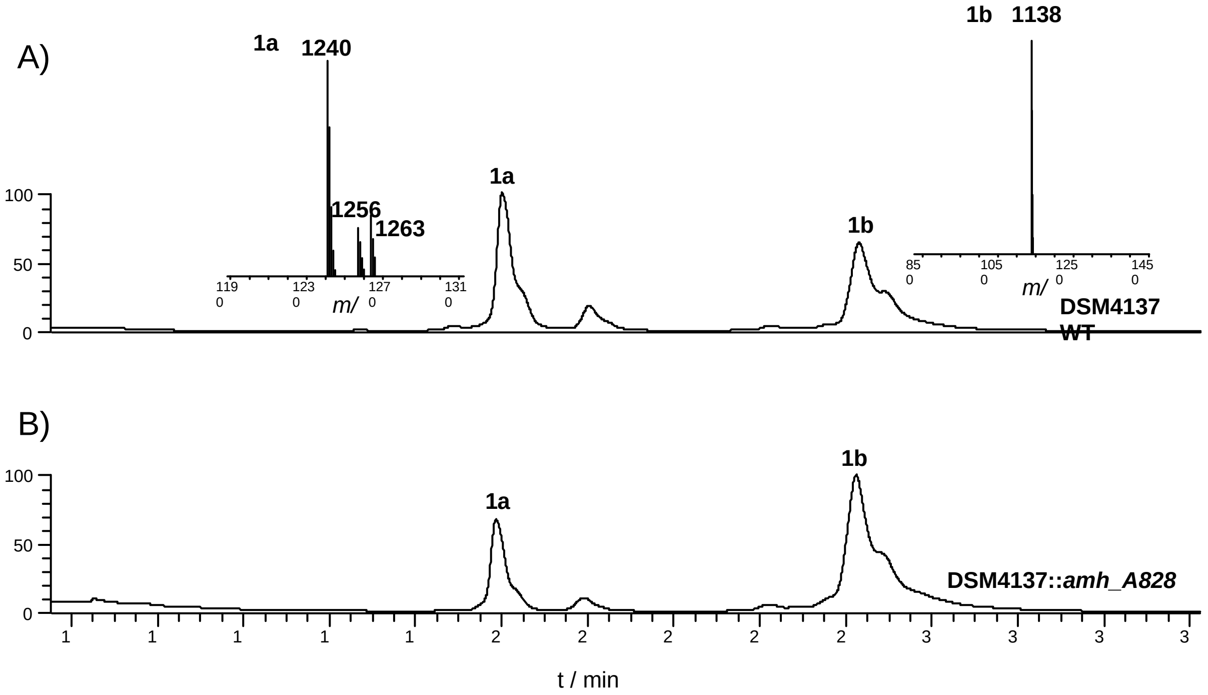

Figure S6: HPLC–UV–MS analysis of polyenes. A) LC–UV (360 nm) trace of methanol extract from mycelium of DSM4137 wild type, showing the production of clethramycin (**1a**) and desulfoclethramycin (**1b**) at *m/z* 1240.8 ([M + Na]$^+$) and 1138.8 ([M + H]$^+$) , respectively. B) LC–UV (360 nm) trace of methanol extract from mycelium of DSM4137 complemented with the amidinohydrolase-encoding gene *amh_A828* from the marginolactone desertomycin biosynthesis from *S. olivaceus* Tü4018 [5], showing that this enzyme was not able to convert **1a** and **1b** to their amino forms.

3. Supplementary Tables

Table S1: Bacterial strains used in this study.

Strain	Genotype/Characteristics	Reference
E. coli		
DH10B	F$^-$ mcrA Δ(*mrr-hsd*RMS - *mcr*BC),<br>□80*lac*ZΔM15, Δ l*ac*X74 *rec*A1 *end*A1 *ara*D139<br>Δ (*ara leu*)7697 *gal*U *gal*K *rpsL nup*G λ–<br>host for general cloning	Invitrogen
BL21(DE3)	F$^-$ ompT hsdS$_B$(rB$^-$, mB$^-$) gal dcm (λDE3 lysogen)<br>host for protein expression	Invitrogen
ET12567 (pUZ8002)	(F$^-$ *dam*-13::Tn9 *dcm*-6 *hsd*M *hsd*R recF143<br>zjj202::Tn10 *gal*K2 *gal*T22 *ara*14 *pac*Y1 *xyl*-5<br>*leu*B6 *thi*-1)<br>Donor strain for conjugation between *E. coli* and<br>*Streptomyces*	[6]

***S. malaysiensis* DSM4137**		
WT-DSM4137	wild type strain, producing azalomycin, desulfo clethramycin, and clethramycin	[7]
□smala2697	*smala2697* in-frame deletion mutant from DSM4137	this work
□smala2697::*smala2697*	□smala2697 mutant complemented with plasmid pIB139-slfV	this work
□smala2697::*medi5536*	□smala2697 mutant complemented with plasmid pIB139-slfM	
DSM4137::*medi4948*	DSM4137 complemented with plasmid pIB139medi4948	this work
DSM4137::*amh-A828*	DSM4137 complemented with plasmid pIB139amh828	this work
		this work
***S. mediocidicus* ATCC23936**	mediomycin- and clethramycin-producing strain	[8]

Table S2: Plasmids used in this work.

Plasmid	Genotype/Characteristics	Reference
pYH7	*E.coli-Streptomyces* shuttle vector	[9]
pYH7-slfV	*smala2697* gene disruption construct in which a 948 bp internal fragment of *smala2697* was deleted inframe	this work
pIB139	*E.coli-Streptomyces* shuttle vector, *attP* (ΦC31), *int*, P_{ermE*}	
pIB139-medi4948	Amidinohydrolase *medi4948* complementation plasmid	This work
pIB139-amh828	Amidinohydrolase *amh-A828* complementation plasmid	This work
pIB139-smala2697	Sulfotransferase *smala2697* complementation plasmid	This work
pIB139-medi5536	Sulfotransferase *medi5536* complementation plasmid	This work
pET28a(+)	*E. coli* protein expression vector	Invitrogen
pET28a-smala2697	Sulfotransferase SMALA_2697 protein expression construct with N-terminal His-tag based on pET28a(+)	this work
pET28a-medi0234	Medi0234 protein expression construct with Nterminal His-tag based on pET28a(+)	this work
pET28a-medi2865	Medi2865 protein expression construct with Nterminal His-tag based on pET28a(+)	this work
pET28a-medi4948	Medi4948 protein expression construct with Nterminal His-tag based on pET28a(+)	this work

Table S3: Oligonucleotide primers used in this work.

Primer	Nucleotide sequence (5' to 3')	Restriction site(s)
primers for protein expression		
medi0234-fwd	tttt CATATG ACGATCCCAGCCACGCCCGG	*Nde*I
medi0234-rev	agctga AAGCTT TCACGACGGCACCCCTCCGT	*Hind*III
medi2865-fwd	tttt CATATG AGCACCACCCCCGCCCCCCG	*Nde*I
medi2865-rev	agctga AAGCTT TCAGTCGCGAGCGGCCGCGA	*Hind*III
medi4948-fwd	tttt CATATG ACGTTCCCCAACGACAAGAC	*Nde*I
medi4948-rev	AAGCTT TCAGGGCTTGCTCCAGTAGG	*Hind*III
smala2697-fwd	TTTTCATATG GTCAACCAGAAGTTGACATT	*Nde*I
smala2697-rev	AGCTGAAAGCTT CTACTGGTGGACGGCCAGCG	*Hind*III
primers for smala2697 gene in-frame deletion		
smala2697-L1	TGATCAAGGCGAATACTTCATATG<br>TGCTGTAACGGTCGGCCATCTGTA	
smala2697-L2	CAGCGAGGTGAG GGTGCCGACGACAAATGTCAACTT	
smala2697-R1	GTCGTCGGCACC CTCACCTCGCTGCTGGAGTCCTGT	
smala2697-R2	CCGCGCGGTCGATCCCCGCATATG<br>TGACCGTCTTCATCGGCGAGAACG	
primers for PCR screening of deletion mutants		
smala2697-CP1	ATGACCGTCTCGTCACAGGA	
smala2697-CP2	GCACACCACGATGATAGGCA	
NdeI-L	GCTCAGGGCGACACGATC	
NdeI-R	CTGACCGGCAATCACCAAC	
primers for smala2697 gene complementation		
smala2697_com_F	AATCGTGCCGGTTGGTAGGATCCACATATGGTGGTC<br>AACCAGAAGTTGAC	
smala2697_com_R	ACAGGAAACAGCTATGACATGATTACGAATTCGATA<br>TCCTACTGGTGGACGGCCAG	
primers for medi5536 gene complementation		
medi5536_com_F	GTGCCGGTTGGTAGGATCCACATATGCACACGGATA<br>AGTTGACCTTTG	
medi5536_com_R	TGACATGATTACGAATTCGATATCCTACAGCCCCTG<br>GCCCGCCAGTG	
Primers for PCR screening and sequencing of complementation mutants		
pIB-seqF	GATCTTGACGGCTGGCGAG	
pIB-seqR	CACTCATTAGGCACCCCAGG	
primers for medi4948 gene complementation		
medi4948_com_F	tttt CATATG ACGTTCCCCAACGACAAGAC	*Nde*I
medi4948_com_R	agctga GATATC TCAGGGCTTGCTC	*Eco*RV
primers for amh_A828 gene complementation		
amh828_com_F	tttt CATATG AGCGAGACACCCGAGTCCGA	*Nde*I
amh828_com_R	agctga GATATC TCACTTGAGCGGGAAGCGCA	*Eco*RV

Table S4a: Properties of genes within the clethramycin biosynthetic gene cluster of *Streptomyces* sp. DSM4137

ORF	Product size (aa)	% identity/ similarity	Species	Putative Function	Database entry
smala2696R	311	96/98	*Streptomyces iranensis*	LysR regulator	WP_044575084
smala2697R	347	84/90	*Streptomyces rapamycinicus*	sulfotransferase	AGP57770.1
smala2698	882	95/97	*Streptomyces violaceusniger*	LuxR regulator	AEM87305.1
smala2699	552	93/95	*Streptomyces rapamycinicus*	arginine oxidase	AGP57768.1
smala2700R	469	96/97	*Streptomyces violaceusniger*	acyl-CoA ligase	AEM87307.1
smala2701R	207	93/97	*Streptomyces violaceusniger*	TEII	AEM87308.1
smala2702	326	96/98	*Streptomyces violaceusniger*	ABC transporter	AEM87309.1
smala2703	477	84/88	*Streptomyces violaceusniger*	ABC transporter	AEM87310.1
smala2704	312	96/97	*Streptomyces rapamycinicus*	ACP:malonyl transferase	AGP57763.1
smala2705R	199	94/96	*Streptomyces rapamycinicus*	TetR regulator	AGP61306.1
smala2706	304	95/97	*Streptomyces rapamycinicus*	□,□-hydrolase	AGP61305.1
smala2707R	907	90/92	*Streptomyces rapamycinicus*	TetR regulator	AGP58152.1
smala2708	248	96/98	*Streptomyces* sp.	short chain dehydrogenase	AGP58153.1
smala2709R	144	93/97	*Streptomyces* sp.	glycosyltransferase	WP_030771662
smala2710R	167	99/100	*Streptomyces scabiei*	bacteriocin biosynthesis protein	KFG10609.1
smala2711R	353	65/74	*Streptomyces sclerotialus*	lanthionine synthetase	WP_030569306
smala2712R	1012	64/75	*Streptomyces scabiei*	lantibiotic dehydratase	KFF98219.1
smala2713R	54	—	*Streptomyces* sp. PRh5	putative lantibiotic precursor	—
smala2714R	400	74/85	*Streptomyces* sp. FxanaA7	protein-Lisoaspartate Omethyltransferase	WP_045558093

smala2715R	287	57/69	*Streptomyces viridochromogenes*	taurine dioxygenase	AFV30253.1
smala2716	155	83/92	*Streptomyces iakyrus*	NUDIX hydrolase	WP_033313602
smala2717	389	73/83	*Streptomyces viridochromogenes*	Xre regulator	ELS55765.1
smala2718R	69	—	—	—	—
smala2719R	219	81/90	*Streptomyces* sp. NTK 937	DNA binding protein	KDQ67008.
smala2720	106	—	—	—	—
smala2721	71	—	—	—	—
smala2722R	3902	91/93	*Streptomyces iranenis*	PKS CleA9	CDR09758.1
smala2723R	5783	92/95	*Streptomyces violaceusniger*	PKS CleA8	AEM87318.1
smala2724R	3206	93/96	*Streptomyces* sp. PRh5	PKS CleA7	EXU62495.1
smala2725R	7345	87/90	*Streptomyces violaceusniger*	PKS CleA6	AEM87320.1
smala2726R	5250	91/94	*Streptomyces violaceusniger*	PKS CleA5	AEM87321.1
smala2727R	1664	93/95	*Streptomyces* sp. PRh5	PKS CleA4	EXU62661.1
smala2728R	8599	91/93	*Streptomyces violaceusniger*	PKS CleA3	AEM87323.1
smala2729R	3444	90/93	*Streptomyces iranensis*	PKS CleA2	CDR09746.1
smala2730R	8207	90/93	*Streptomyces* sp. PRh5	PKS CleA1	EXU66032.1
smala2731R	514	91/96	*Streptomyces rapamycinicus*	membrane protein	AGP57745.1
smala2732R	166	96/98	*Streptomyces rapamycinicus*	membrane protein	AGP57744.1
smala2733R	185	68/82	*Streptomyces sp. 769*	membrane protein	AJC60945.1
smala2734	414	93/96	*Streptomyces rapamycinicus*	sensor kinase	AGP57742.1
smala2735	186	96/99	*Streptomyces rapamycinicus*	LuxR regulator	AGP57741.1
smala2736R	851	93/95	*Streptomyces rapamycinicus*	LuxR regulator	AGP57740.1
smala2737R	253	93/94	*Streptomyces rapamycinicus*	TEII thioesterase	AGP57739.1

Putative functions of the encoded proteins were deduced from analyses with the BlastP program (http://blast.ncbi.nlm.nih.gov/Blast.cgi). The % identity/similarity for the protein in the database with the highest end-to-end similarity is indicated. *R* designates a gene lying on the opposite strand. The entire genome sequence of *S. malaysiensis* DSM4137 has been deposited in GenBank where it can be accessed as *S. malaysiensis* Bioproject PRJNA396489, Biosample SAMN07427119.

Table S4b: Properties of genes within the mediomycin biosynthetic gene cluster of *Streptomyces mediocidicus* ATCC 23936

ORF	Product size (aa)	% identity/ similarity	Species	Putative Function	Database entry
orf5537	210	54/66	*Nocardiopsis* sp. NRRL B16309	permease	WP_053619846
medi5536	349	96/98	*Streptomyces blastmyceticus*	sulfotransferase	BAM21064.1
medi5535R	942	95/97	*Streptomyces blastmyceticus*	LuxR regulator	BAM21065.1
medi5534R	553	97/98	*Streptomyces blastmyceticus*	arginine oxidase	BAM21066.1
medi5533	468	83/90	*Streptomyces violaceusniger*	acyl-CoA ligase	AEM87307.1
medi5532	213	80/86	*Streptomyces hygroscopicus*	thioesterase TEII	WP_030843507
medi5531R	314	80/87	*Streptomyces hygroscopicus*	ACP:malonyl transferase	WP_030843518
medi5530	3833	77/84	*Streptomyces violaceusniger*	PKS medA9	AEM87317.1
medi5529	5702	79/86	*Streptomyces rapamycinicus*	PKS medA8	AGP57754.1
medi5528	3212	82/89	*Streptomyces himastatinicus*	PKS medA7	EFL26042.1
medi5527	7131	79/85	*Streptomyces violaceusniger*	PKS medA6	AEM87320.1
medi5526	5163	78/85	*Streptomyces violaceusniger*	PKS medA5	AEM87321.1
medi5525	1651	79/87	*Streptomyces* sp. PRh5	PKS medA4	EXU62661.1
medi5524	8399	77/83	*Streptomyces violaceusniger*	PKS medA3	AEM87320.1
medi5523	3377	76/83	*Streptomyces* sp. PRh5	PKS medA2	EXU66033.1
medi5522	8123	76/83	*Streptomyces* sp. PRh5	PKS medA1	EXU66032.1
medi5521	518	70/81	*Streptomyces rapamycinicus*	membrane protein	AGP57745.1
medi5520	151	85/91	*Streptomyces iranensis*	membrane protein	CDR09741.1
medi5519	177	60/77	*Streptomyces aizunensis*	membrane protein	AAX98180.1
medi5518R	417	73/83	*Kitasatospora mediocidica*	sensor histidine kinase	WP_035796319

medi5517R	201	88/93	*Streptomyces hygroscopicus*	LuxR regulator	WP_051886463
medi5516	963	67/78	*Streptomyces hygroscopicus*	LuxR regulator	WP_030836742
medi5515	216	78/86	*Streptomyces himastatinicus*	TEII thioesterase	EFL26014.1

Putative functions of the encoded proteins were deduced from analyses with the BlastP program (http://blast.ncbi.nlm.nih.gov/Blast.cgi). The % identity/similarity for the protein in the database with the highest end-to-end similarity is indicated. The entire genome sequence of *S. mediocidicus* ATCC23936 has been deposited in GenBank where it has been classified as *S. blastmyceticus* and can be accessed as *S. blastmyceticus* Bioproject PRJNA411827, Biosample SAAMN07688521.

4. Supplementary References

[1] Sambrook, J. Russell, D W. Molecular Cloning: A Laboratory Manual, 3rd ed. Cold Spring Harbor Laboratory Press, New York, **2001**.

[2] Kieser, T, Bibb, M, Buttner, M, Chater, K F, Hopwood, D A, Practical *Streptomyces Genetics*. The John Innes Foundation, Norwich, **2001**.

[3] Gibson, D. G.; Young, L.;. Chuang, R. Y.; Venter, J. C.; Hutchison, C. A. III.; Smith, H. O. *Nat. Methods* **2009**, *6*, 343–345.

[4] Zhang, L.; Hashimoto, T.; Qin, B.; Hashimoto, J.; Kozone, I.; Kawahara, T.; Okada, M.; Awakawa, T.; Ito, T.; Asakawa, Y.; Ueki, M.; Takahashi, S.; Osada, H.; Wakimoto, T.; Ikeda, H.; Shin-Ya, K.; Abe, I. *Angew. Chem. Int. Ed.* **2017**, *56*,1740–1745.

[5] Hong, H.; Samborskyy, M.; Lindner, F.; Leadlay, P F. *Angew. Chem. Int. Ed.* **2016**, *55*, 1118–1123.

[6] MacNeil, D. J.; Gewain, K. M.; Ruby, C. L.; Dezeny, G.; Gibbons, P. H.; MacNeil, T. *Gene* **1992**, *111*, 61–68.

[7] Hong, H.; Fill, T.; Leadlay, P. F. *Angew. Chem. Int. Ed.* **2013**, *52*, 13096–13099.

[8] Cai, P.; Kong, F.; Fink, P.; Ruppen, M. E.; Williamson, R. T.; Keiko, T. *J. Nat. Prod.* **2007**, *70*, 215–219.

[9] Sun, Y.; Hahn, F.; Demydchuk, Y.; Chettle, M.; Tosin, M.;. Osada, H.; Leadlay, P. F. *Nat. Chem. Biol.* **2010**, *6*, 99–101.
